# Asciminib monotherapy in patients with chronic myeloid leukemia in chronic phase without *BCR::ABL1*^T315I^ treated with at least 2 prior TKIs: Phase 1 final results

**DOI:** 10.1038/s41375-025-02578-7

**Published:** 2025-04-09

**Authors:** Andreas Hochhaus, Dong-Wook Kim, Jorge E. Cortes, Koji Sasaki, Michael J. Mauro, Timothy P. Hughes, Massimo Breccia, Moshe Talpaz, Hironobu Minami, Yeow Tee Goh, Daniel J. DeAngelo, Fabian Lang, Oliver Ottmann, Michael C. Heinrich, Valle Gomez Garcia de Soria, Philipp le Coutre, Gessami Sanchez-Olle, Meng Cao, Nathalie Pognan, Shruti Kapoor, Matthias Hoch, Delphine Rea

**Affiliations:** 1 Hematology/Oncology, Universitätsklinikum Jena, Jena, Germany.; 2 Uijeongbu Eulji Medical Center, Geumo-dong, Uijeongbu-si, South Korea.; 3 Georgia Cancer Center at Augusta University, Augusta, GA, USA.; 4 Department of Leukemia, The University of Texas MD Anderson Cancer Center, Houston, TX, USA.; 5 Memorial Sloan Kettering Cancer Center, New York, NY, USA.; 6 South Australian Health and Medical Research Institute and University of Adelaide, Adelaide, SA, Australia.; 7 Department of Translational and Precision Medicine-Az., Policlinico Umberto I-Sapienza University, Rome, Italy.; 8 Division of Hematology-Oncology, University of Michigan Rogel Cancer Center, Ann Arbor, MI, USA.; 9 Kobe University Hospital, Kobe, Japan.; 10 Department of Haematology, Singapore General Hospital, Bukit Merah, Singapore.; 11 Department of Medical Oncology, Dana-Farber Cancer Institute, Boston, MA, USA.; 12 Department of Medicine, Hematology and Oncology, Goethe University Hospital, Frankfurt am Main, Germany.; 13 Division of Cancer and Genetics, School of Medicine, Cardiff University, Cardiff, UK.; 14 Department of Medicine, Division of Hematology and Oncology, Portland VA Health Care System and Oregon Health & Science University, Knight Cancer Institute, Portland, OR, USA.; 15 Hospital de la Princesa, Madrid, Spain.; 16 Charité-Universitätsmedizin Berlin, Berlin, Germany.; 17 Novartis Pharma AG, Basel, Switzerland.; 18 Global Drug Development, Novartis Pharma S.A.S., Rueil-Malmaison, France.; 19 Novartis Pharmaceuticals Corporation, East Hanover, NJ, USA.; 20 Service d’Hématologie Adulte et CIC, Hôpital Saint-Louis, Paris, France.

## Abstract

Asciminib is the first approved BCR::ABL1 inhibitor that Specifically Targets the ABL Myristoyl Pocket (STAMP). The present final analysis of the phase 1, open-label, nonrandomized trial (NCT02081378) assessed the long-term safety, tolerability, and antileukemic activity of asciminib in 115 patients with chronic myeloid leukemia in chronic phase without the *BCR::ABL1*^T315I^ mutation who received asciminib 10–200 mg twice daily (BID) or 80–200 mg once daily (cutoff: March 14, 2023). Median exposure duration was 5.9 (range, 0–8.4) years; 60.9% of patients continued receiving asciminib through post-trial access. Grade ≥3 adverse events (AEs) occurred in 88 patients (76.5%). AEs led to treatment discontinuation, dose adjustment/interruption, or additional therapy in 15 (13.0%), 74 (64.3%), and 106 (92.2%) patients, respectively. Most first-ever AEs, particularly hematologic AEs, presented within the first year and no new safety signals emerged. Of 56 patients who achieved major molecular response, 50 maintained the response by cutoff; the Kaplan-Meier-estimated probability of maintaining this response for ≥432 weeks (≈ 8.3 years) was 88% (95% confidence interval, 78.2–97.0%). The recommended dose for expansion was determined at 40 mg BID. With up to 8.4 years of treatment, asciminib continued to demonstrate long-term safety and efficacy in this population.

## INTRODUCTION

Tyrosine kinase inhibitors (TKIs) have extended the life expectancy of patients with chronic myeloid leukemia (CML) to close to that of the general population [1–4].

However, TKI resistance and intolerance remain a challenge, often necessitating treatment discontinuation or switch to an alternative therapy [5–11]. Up to 51% of patients with ≥2 prior TKIs switch treatments due to resistance and/or intolerance [11–16], although switching TKIs may not improve adverse events (AEs) in some patients [7, 17]. Resistance is frequently associated with emergent BCR::ABL1 kinase domain mutations, such as *BCR::ABL1*^T315I^, in patients who may have limited available TKI options [2, 4, 18, 19].

Patients with resistance and/or intolerance to ≥ 2 TKIs have a lower probability of favorable long-term outcomes, including molecular responses (MRs) and overall survival [11, 12, 14, 20–23]. Therefore, effective treatments are needed to reduce treatment switching and improve clinical outcomes [12, 20, 21].

Asciminib is the first and only approved BCR::ABL1 inhibitor that Specifically Targets the ABL Myristoyl Pocket (STAMP) to allosterically inhibit ABL1 kinase activity [24–28]. Compared with ATP-competitive TKIs, asciminib has higher specificity for ABL1, minimizing off-target effects and inducing MRs with improved safety and tolerability [15, 24, 25, 28–30]. The first analysis of the first-in-human phase 1 trial (cutoff, September 1, 2017; median follow-up, 14 months) established the safety and tolerability of asciminib in heavily pretreated patients with CML in chronic phase (CP) or accelerated phase (AP) with or without *BCR::ABL1*^T315I^ [30]. Among patients with CML-CP without *BCR::ABL1*^T315I^, major molecular response (MMR [*BCR::ABL1* ≤ 0.1% on the International Scale (IS)]) was achieved or maintained by year 1 in 44 of 91 evaluable patients (48%), demonstrating preliminary efficacy [30]. Results from the previous analysis of this trial in patients with CML-CP without *BCR::ABL1*^T315I^ after 4.2 years’ median exposure (cutoff, January 6, 2021) demonstrated continued MRs with long-term use; 53 patients (61.6%) achieved MMR and 48 of 53 maintained MMR or achieved deeper responses by the cutoff [16]. Furthermore, the cumulative MMR rate increased over time, and additional patients achieved responses even after 3 years of treatment, demonstrating the continued opportunity to benefit from asciminib with long-term use [16]. No new safety signals were identified in this patient population after additional exposure [16, 30].

In a subsequent phase 3 trial, ASCEMBL, which compared asciminib with bosutinib in patients with CML-CP after ≥ 2 TKIs, patients achieved deeper MR with asciminib than with bosutinib [15, 25].

After initial approval in 2021, the US asciminib product label was recently updated; asciminib received accelerated approval for adults with newly diagnosed Philadelphia chromosome–positive (Ph + ) CML-CP, supported by results from the ASC4FIRST trial, and full approval for adults with Ph+ CML-CP that is previously treated or who have *BCR::ABL1*^T315I^, supported by the current phase 1 and ASCEMBL trials [15, 16, 25, 26, 30–32]. Asciminib is approved in > 70 countries for patients with CML-CP after ≥ 2 TKIs and for patients with *BCR::ABL1*^T315I^ in some countries [33]. Reported here is the final analysis from the phase 1 trial (NCT02081378) [34] assessing the long-term safety, tolerability, and antileukemic activity of asciminib monotherapy in patients with CML-CP without *BCR::ABL1*^T315I^ after ≥ 2 TKIs with ≤ 8.4 years of exposure.

## METHODS

### Study oversight

The sponsor (Novartis Pharmaceuticals Corporation) and lead study investigators collaboratively designed the study. The sites’ institutional review boards or ethics committees approved the protocol, which was conducted in accordance with the Declaration of Helsinki. All patients provided written informed consent. The sponsor collected the data and analyzed them in conjunction with the authors. All authors contributed to the development and writing of the manuscript and vouch for the accuracy and completeness of the data and the study’s fidelity to the protocol.

### Study design

The study design has been previously described in detail [16, 30]. Briefly, this was an open-label, nonrandomized, first-in-human study of asciminib as monotherapy and in combination with nilotinib, imatinib, or dasatinib in previously treated patients with Ph+ CML-CP/AP with or without *BCR::ABL1*^T315I^ and as monotherapy in previously treated CML in blast phase or Ph+ acute lymphoblastic leukemia (Fig. S1).

The present analysis includes all patients with Ph+ CML-CP without *BCR::ABL1*^T315I^ at screening who received asciminib monotherapy either twice daily (BID; 10–200 mg) or once daily (QD; 80–200 mg) (Table S1). Additional focus is given in reporting the approved doses of 40 mg BID or 80 mg QD. Patients were ≥18 years of age, previously treated with ≥2 different TKIs prior to study entry, and had relapsed on, were refractory to, or were intolerant of TKIs as determined by the investigator using standard criteria [30, 35].

The primary objective was to determine the maximum tolerated dose and/or recommended dose for expansion for asciminib monotherapy. Secondary objectives included assessing asciminib’s safety and tolerability, its preliminary antileukemic activity, and its pharmacokinetic (PK) profile in plasma. The end of study was declared when all patients enrolled had completed study treatment and all applicable study visits.

### Study assessments

Assessments were performed as previously described [16, 30]. AEs that occurred during the on-treatment period (≤ 30 days after the last dose) were reported. MRs were assessed using real-time quantitative reverse-transcriptase polymerase chain reaction on the first day of cycles 2, 3, 6, 9, and 13 for dose escalation cohorts or 2, 3, 4, 7, 10, and 13 for dose expansion cohorts, every 3 cycles afterwards, and at the end-of-treatment visit. Each cycle was 28 days. Results were reported as the ratio of *BCR::ABL1/ABL1* on the IS [30, 36].

*BCR::ABL1* mutational analyses were performed using Sanger sequencing at screening, end of treatment, upon an unconfirmed loss of response, and/or as clinically indicated. MR and mutational analyses were performed centrally by ICON (Portland, OR, USA). If a mutation was detected, additional analyses were performed every 3 cycles until the mutation was no longer detected or at the discretion of the investigator.

Estimation of maximum tolerated dose and recommended dose for expansion was calculated based on the estimation of the probability of dose-limiting toxicities (DLTs) in cycle 1 for patients in the dose-determining set. PK samples were collected for and evaluated in all patients with CML-CP/AP with or without *BCR::ABL1*^T315I^ at study entry who received asciminib monotherapy at all dose levels.

### Statistical analyses

The final analysis cutoff date was March 14, 2023, and included all patients in this cohort who received ≥1 asciminib dose. Safety and efficacy analyses included patients who received ≥1 asciminib dose (*N* = 115). PK analyses included all patients who had ≥1 blood sample providing an evaluable full PK profile (cycle 1 day 1, cycle 1 day 15, or cycle 2 day 1). MR rates were calculated as the proportion of eligible patients (without atypical *BCR::ABL1* transcripts at screening) who were in a response level at or before (ie, cumulative rate) the specified visit; MR achievement rates excluded patients with the indicated MR level at baseline. Time to MMR was defined as the time between the date of study start and first observation of *BCR::ABL1*^IS^ ≤ 0.1% in patients without MMR and with typical *BCR::ABL1* transcripts at baseline. Duration of first MMR was defined as the time between the date when *BCR::ABL1*^IS^ ≤ 0.1% was first observed and the date of confirmed loss of MMR before the cutoff date, estimated in MMR-evaluable patients using the Kaplan-Meier method.

## RESULTS

### Patients

This analysis included 115 patients with CML-CP without *BCR::ABL1*^T315I^ who were enrolled in the study and received asciminib monotherapy across all starting doses (Table S1). The median age of patients was 56.0 (25–88) years, with 73.9% of patients < 65 years old (Table S2) [16]. Most patients (71.3%) had received ≥3 prior TKIs; prior TKIs included dasatinib (*n* = 98; 85.2%), nilotinib (*n* = 89; 77.4%), imatinib (*n* = 85; 73.9%), bosutinib (*n* = 45; 39.1%), and/or ponatinib (*n* = 36; 31.3%) [16]. Most commonly used TKIs were imatinib (56%) as a first TKI, dasatinib (38% and 30%) and nilotinib (34% and 16%) as second or third TKIs, respectively, bosutinib (17%) as a fourth TKI, and ponatinib (7%) as a fifth TKI [16]. Three patients had *BCR::ABL1*^T315I^ detected at enrollment and had received 1 prior TKI per eligibility criteria for patients with *BCR::ABL1*^T315I^; the mutation was not confirmed by the central laboratory [16].

At screening, most (*n* = 86; 74.8%) patients had *BCR::ABL1*^IS^ > 0.1%; 20 (17.4%) were in MMR, and 5 were in deep molecular response (DMR); 9 (7.8%) patients had atypical/e1a2/unknown *BCR::ABL1* transcripts [16]. *BCR::ABL1* mutations were detected in 12 patients (10.4%) at screening; 10 of these had only 1 mutation, including F317L (*n* = 3), E255K (*n* = 2), and G250E, L248V, M244V, V299L, and Y253H (*n* = 1 each) [16]. Two of 12 patients had multiple mutations detected (G250E, L248V, and V299L [*n* = 1], and G250E and M244V [*n* = 1]) [16].

**Fig. 1:**
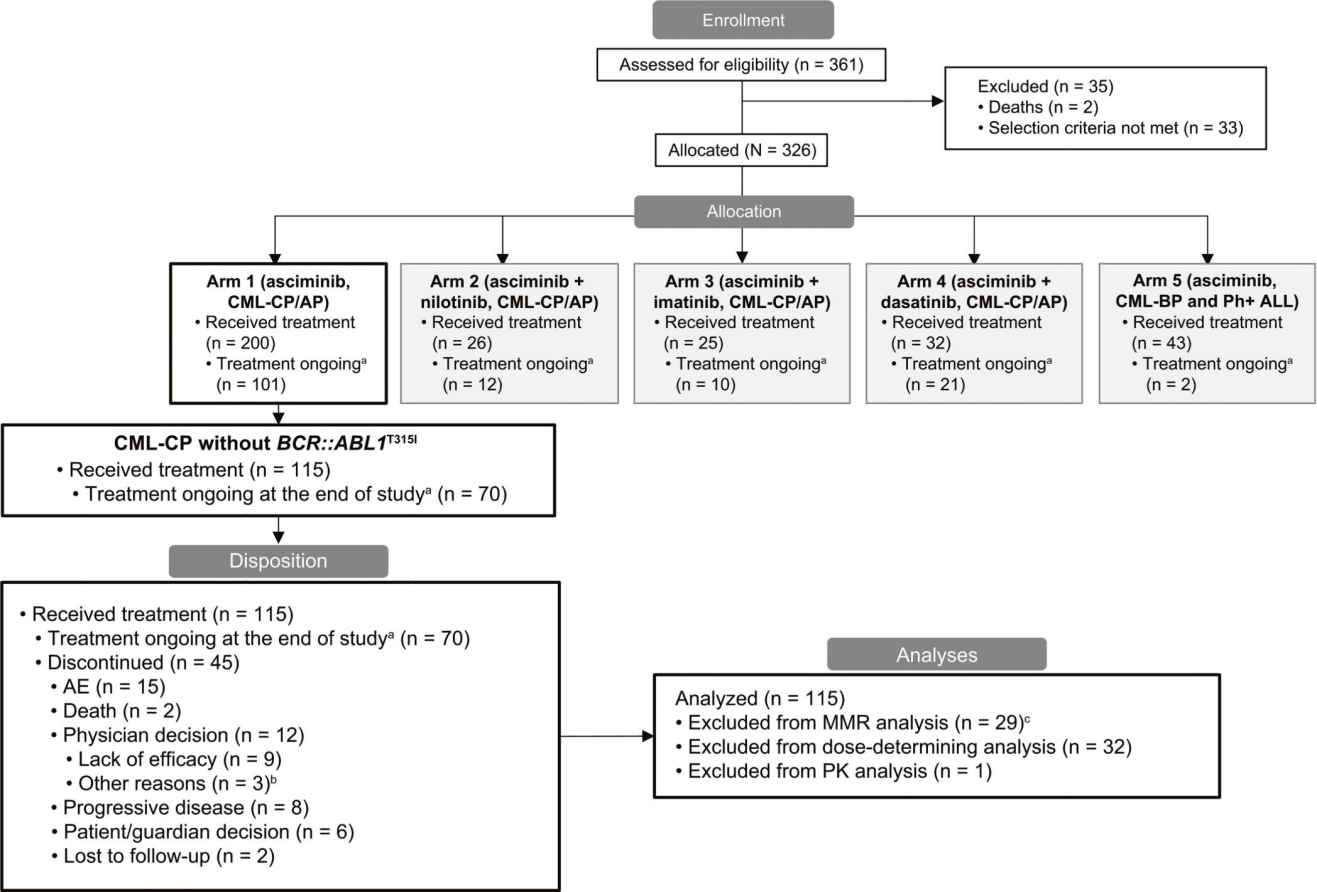
Patient disposition as of the data cutoff (March 14, 2023) and analysis groups. Disposition and analysis sets include patients with CML-CP without *BCR::ABL1*^T315I^ mutations who received asciminib monotherapy. AE adverse event, ALL acute lymphoblastic leukemia, AP accelerated phase, BP blast phase, CML chronic myeloid leukemia, CP chronic phase, IS International Scale, MMR major molecular response (*BCR::ABL1*^IS^ ≤ 0.1%); MR^4^, *BCR::ABL1*^IS^ ≤ 0.01%; MR^4.5^, *BCR::ABL1*^IS^ ≤ 0.0032%; Ph + Philadelphia chromosome positive, PK pharmacokinetic, TFR treatment-free remission. ^a^Treatment ongoing as of the cutoff date (March 14, 2023) in post-trial access, including patients who continued to receive asciminib in a rollover study or commercial availability. ^b^Other reasons included treatment free remission attempt (*n* = 2) and other comorbidities (*n* = 1). ^c^Nine patients were excluded due to having atypical or unknown *BCR::ABL1* transcripts at screening. From Mauro MJ, et al. Leukemia. 2023;37:1048–59 [16].

By the cutoff, 45 patients (39.1%) had discontinued treatment, predominantly due to AEs (*n* = 15; 13%), physician decision due to lack of efficacy (*n* = 9; 7.8%), and progressive disease (*n* = 8; 7.0%) (Fig. 1). Patient disposition by the starting dose of study treatment is reported in Table S3. Seventy patients (60.9%) continued receiving asciminib after the final analysis through post-trial access, including, but not limited to, the rollover study and reimbursable commercial supply.

The median duration of asciminib exposure was 5.9 years (range, 0–8.4 years); 91 patients (79.1%) had at least 96 weeks (≈ 2 years) of exposure and 85 (73.9%) had at least 144 weeks (≈ 3 years) of exposure. Patients who received 40-mg BID and 80-mg QD starting doses had a median duration of exposure of 6.4 years (range, 0–8.4 years) and 5.6 years (range, 0.3–6.7 years), respectively. Among evaluable patients, most remained on asciminib for longer than their last prior TKI, regardless of the number of prior TKIs received (Fig. S2).

### Safety

Treatment-emergent all-grade AEs occurred in all patients; most were grade 1/2 (Table 1). The most common grade ≥3 AEs (≥10%) were increased lipase (21.7%), arterial hypertension (18.3%), and thrombocytopenia (10.4%). In the 40-mg BID and 80-mg QD starting-dose cohorts, all-grade and grade ≥ 3 AEs were mostly comparable (within ≤ 15% difference) (Table S4).

**Fig. 2:**
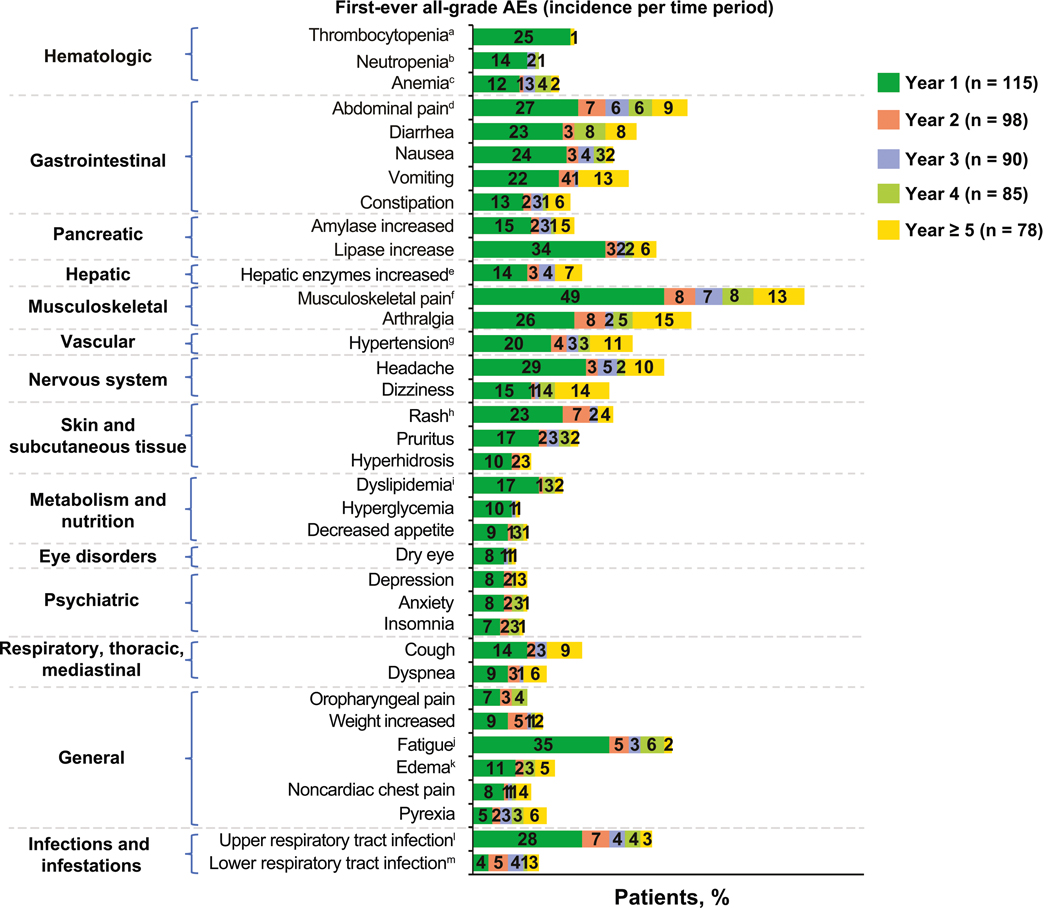
First-ever all-grade adverse events (incidence) by year of asciminib treatment. AE adverse event, PT preferred term. Proportions were calculated based on the number of patients at risk of an event (patients ongoing treatment and event free at the start of the interval). The number of patients at risk of an event differed from year to year, and percentages in each year should thus not be summed. A patient with multiple occurrences of an event within the same time interval was counted only once in that time interval. A patient who experienced an ongoing or recurrent AE over multiple years of asciminib treatment was counted in multiple time periods. The safety topics corresponded to either single PTs or groups of PTs according to the adverse drug reaction definitions. ^a^Thrombocytopenia included PTs thrombocytopenia and decreased platelet count. ^b^Neutropenia included PTs neutropenia and neutrophil count decreased. ^c^Anemia included PTs anemia and decreased hemoglobin. ^d^Abdominal pain included PTs abdominal pain and upper abdominal pain. ^e^Hepatic enzymes increased included PTs increased alanine aminotransferase, increased γ-glutamyltransferase, increased aspartate aminotransferase, and transaminases increased. ^f^Musculoskeletal pain included PTs pain in extremity, myalgia, back pain, bone pain, neck pain, musculoskeletal pain, musculoskeletal chest pain, and musculoskeletal discomfort. ^g^Hypertension included PTs increased blood pressure and hypertension. ^h^Rash included PTs rash and maculopapular rash. ^i^Dyslipidemia included PTs increased blood cholesterol, increased blood triglycerides, hypertriglyceridemia, hypercholesterolemia, and hyperlipidemia. ^j^Fatigue included PTs fatigue and asthenia. ^k^Edema included PTs edema and peripheral edema. ^l^Upper respiratory tract infection includes PTs upper respiratory tract infection, nasopharyngitis, pharyngitis, and rhinitis. ^m^Lower respiratory tract infection included PTs pneumonia and bronchitis.

Observing the occurrence of first-ever AEs (incidence) over time, most AEs presented within the first year of treatment (Fig. 2). Particularly with hematologic AEs, few patients experienced a first-ever event after the first year. No new safety signals emerged with > 5 years’ median exposure.

Lipase increase (*n* = 4), and amylase increase and thrombocytopenia (*n* = 2 each), were the most frequent AEs leading to treatment discontinuation (Table S5). Since the previous analysis, 2 additional patients had AEs leading to discontinuation (myalgia, *n* = 1; neutropenia and thrombocytopenia, *n* = 1) [16].

AEs leading to dose adjustment or interruption occurred in 74 patients (64.3%); this rate was similar to that seen in the previous analysis [16], with 5 new patients requiring dose adjustment or temporary interruption per protocol; events suspected to be related to study treatment are listed in Table S6. The most frequent AEs requiring additional therapy (≥ 10%) were arthralgia (*n* = 29; 25.2%), upper respiratory tract infection (*n* = 27; 23.5%), headache (*n* = 23; 20.0%), arterial hypertension (*n* = 21; 18.3%), nausea and back pain (*n* = 16; 13.9% each), constipation and rash (*n* = 15; 13.0% each), and anemia (*n* = 12; 10.4%).

The most frequent all-grade AEs of special interest (AESIs; ≥ 20%) were gastrointestinal events (*n* = 85; 73.9%), hypersensitivity (*n* = 56; 48.7%), pancreatic events (including enzyme elevations; *n* = 54; 47.0%), myelosuppression (including anemia, leukopenia, thrombocytopenia, and cytopenias affecting > 1 lineage; *n* = 42; 36.5%), hepatic events (including elevations of liver enzymes and/or bilirubin, and decreased albumin; *n* = 36; 31.3%), edema and fluid retention (*n* = 35; 30.4%), and hemorrhage (*n* = 27; 23.5%). In the 40-mg BID and 80-mg QD starting-dose cohorts, AESI rates were mostly similar (within ≤ 15% difference) to those of the entire study population (Table S7). The exposure-adjusted incidence rates for all-grade AESIs are reported in Table 2.

Gastrointestinal events occurred in 85 patients, including 2 new patients since the previous analysis [16]; 1 of these 2 patients had a grade 3 event. Most of these events were grade 1/2; few required dose adjustment (*n* = 9; 7.8%) or interruption (*n* = 11; 9.6%), and none led to treatment discontinuation.

Hypersensitivity occurred in 56 patients, including 5 new patients since the previous analysis [16]; the most common events (>5%) were rash (25.2%), maculopapular rash (6.1%), and urticaria (6.1%). Most of these events, including all new events, were grade 1/2, and no new patients required dose adjustments or interruptions beyond those previously reported [16]. As previously reported, 1 patient in the 200 mg BID cohort had grade 1 rash and grade 3 bronchospasm leading to treatment discontinuation, along with grade 3 cyanosis [16].

Pancreatic events, including clinical pancreatitis and increased lipase and amylase activities, occurred in 54 patients, 4 of which were new from the previous analysis; grade ≥3 events occurred in 32 patients, 2 of which were new from the previous analysis. Most pancreatic events resolved by the cutoff date; 6 patients (5.2%) required concomitant medication, 6 (5.2%) discontinued treatment, and 16 (13.9%) and 22 (19.1%) required dose adjustment or interruption, respectively. Eight patients had clinical pancreatitis (grade 3, *n* = 4; grade 4, *n* = 0); these events led to treatment discontinuation (*n* = 2), dose adjustment (*n* = 3), and/or temporary interruption of treatment (*n* = 5). By cutoff, most clinical pancreatitis events had resolved. No additional patients since the previous cutoff date experienced clinical pancreatitis [16].

Myelosuppression occurred in 42 patients, including 2 new events since the previous analysis; grade ≥3 events occurred in 23 patients [16]. The most frequent myelosuppression events were thrombocytopenia (*n* = 30), anemia (*n* = 21), and neutropenia (*n* = 19), consistent with the previous analysis [16]. Since the previous analysis, 1 additional patient discontinued treatment due to myelosuppression events (decreased neutrophil count and thrombocytopenia); 2 other patients discontinued treatment due to thrombocytopenia.

Arterial occlusive events (AOEs) occurred in 14 patients (12.2%); when adjusted for patient-year exposure, the incidence was 2.6% (Tables S7 and S8). Six patients experienced grade 3 events, and 1 patient experienced grade 4 cerebrovascular accident and also had grade 2 angina pectoris and grade 1 troponin T increase; no fatal events occurred. Four new patients had AOEs since the previous analysis [16]. One patient had angina pectoris (grade 1); they had previously received nilotinib, imatinib, and dasatinib, and their relevant active conditions included type II diabetes mellitus, arterial hypertension, hyperlipidemia, renal failure, and chronic kidney disease. Another patient had angina pectoris (grade 2); they had previously received nilotinib, imatinib, and dasatinib; no relevant prior history was noted, and active conditions included hypertension, chronic kidney disease, and increased blood creatinine. Another patient had carotid artery stenosis; they had previously received nilotinib, dasatinib, and ponatinib and had no relevant history but did have arterial hypertension as a relevant active condition. Another patient had angina pectoris, cerebrovascular accident, and troponin T increase; they had previously received imatinib, nilotinib, dasatinib, and bosutinib; relevant prior history included deep vein thrombosis, and active conditions included obesity, cardiomyopathy, arterial hypertension, asthma, chronic bronchitis, and hyperlipidemia. AOEs led to dose adjustment in 3 patients and interruption in 4 patients; none led to treatment discontinuation. No new on-treatment deaths occurred since the previous analysis [16].

### Efficacy

**Fig. 3:**
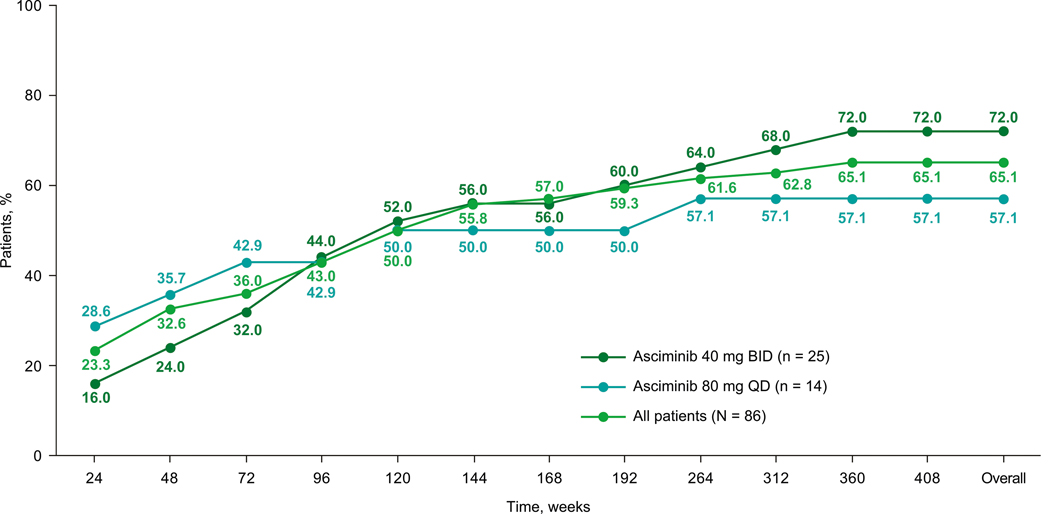
Cumulative MMR rate in evaluable^a^ patients who were not in MMR at screening by starting dose. BID twice daily, IS International Scale, MMR major molecular response (*BCR::ABL1*^IS^ ≤ 0.1%); QD once daily. ^a^Excluded patients who had atypical or unknown transcripts at screening.

All MR analyses excluded patients with atypical, e1a2, or unknown *BCR::ABL1* transcripts at baseline (*n* = 9). Among evaluable patients (*N* = 106), 76.4% were in *BCR::ABL1*^IS^ ≤ 1% by week 264, with responses observed regardless of starting dose (Fig. S3). The assessment of cumulative MMR achievement additionally excluded patients with MMR or deeper responses at baseline (*n* = 20); of the 86 MMR-evaluable patients, 56 (65.1%) achieved MMR by the cutoff date (Fig. 3), with half having done so by week 48 (year ≈ 1). The MMR achievement rate continued to increase over the treatment period, with patients still achieving their first MMR up to year 6.9 (median time to MMR: 58.3 weeks [range, 2–360 weeks]). With longer follow-up, 50 of 56 patients (89.3%) who achieved MMR maintained or achieved deeper responses by the cutoff. Five of the 6 patients who lost MMR were previously reported [16]. The newly reported patient had received treatment for 7.2 years and was in *BCR::ABL1*^*I*S^ ≤ 1% at the cutoff; this patient continued receiving asciminib via post-trial access.

The Kaplan-Meier-estimated proportion of patients maintaining their first MMR for at least 96 weeks (1.8 years) was 92% (95% CI, 85.3–99.6%); thereafter, the proportion of patients maintaining their first MMR ranged from 90% (95% CI, 82.2–98.4%) at week 120 (2.3 years) to 88% (95% CI, 78.2–97.0%) at week 432 (8.3 years).

**Fig. 4:**
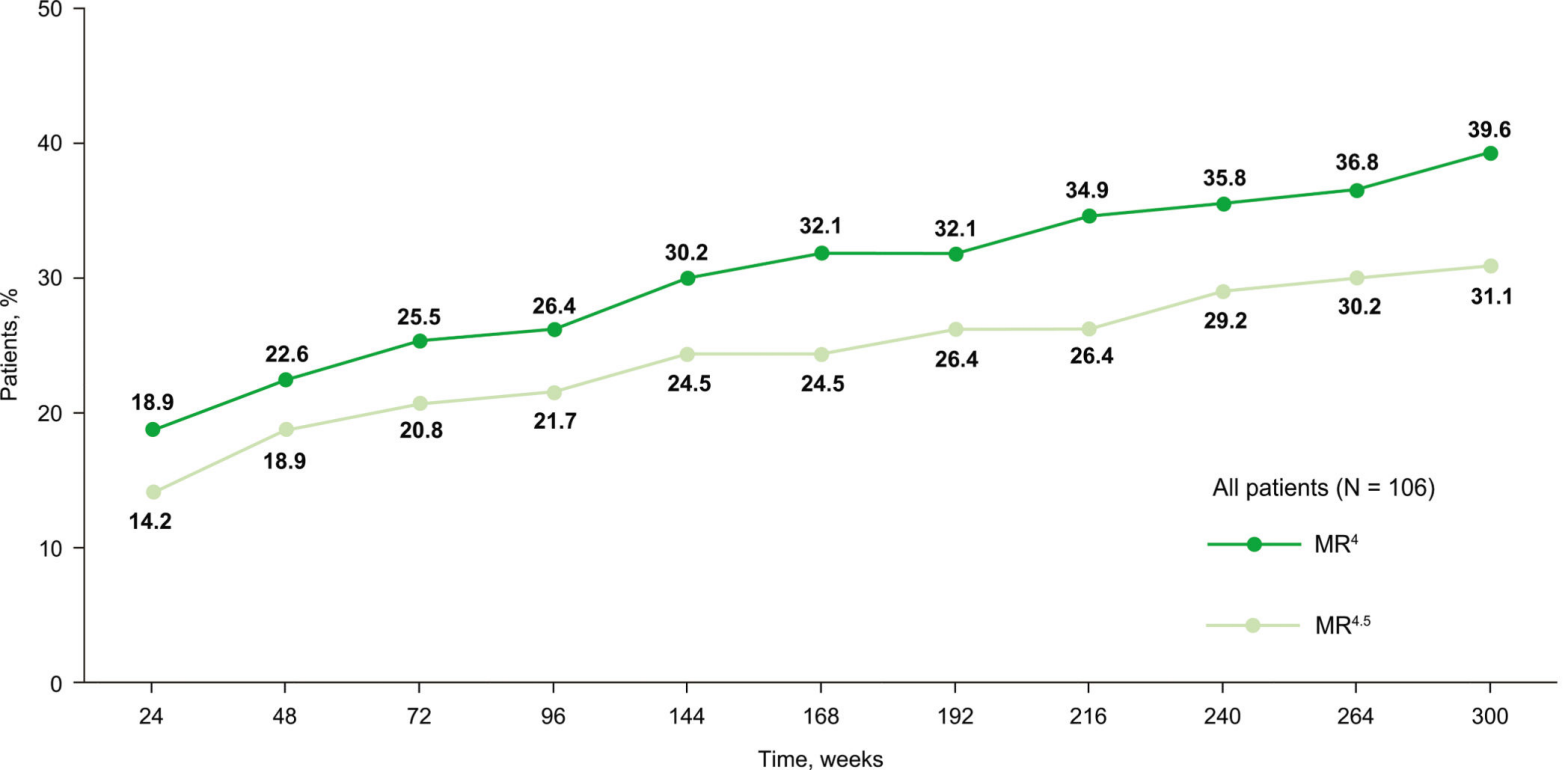
Cumulative rates of deep molecular responses (MR^4^ or MR^4.5^) in evaluable^a^ patients. MMR major molecular response, MR^4^, *BCR::ABL1*^IS^ ≤ 0.01%; MR^4.5^, *BCR::ABL1*^IS^ ≤ 0.0032%. ^a^Included all patients with evaluable transcripts not expressing an atypical or e1a2 or unknown transcript and not having a missing evaluation at screening.

DMR rates increased over time with MR^4^ (*BCR::ABL1*^IS^ ≤ 0.01%) increasing from 22.6% by week 48 to 39.6% by week 300 and MR^4.5^ (*BCR::ABL1*^IS^ ≤ 0.0032%) increasing from 18.9% by week 48 to 31.1% by week 300 (Fig. 4). MMR and MR^4.5^ rates increased at each time point reported up to week 144 (Table S9). Cumulative rates of *BCR::ABL1*^IS^ ≤ 10%, *BCR::ABL1*^IS^ ≤ 1%, MMR, and DMR increased through week 48, 120, 288, and 300, respectively (Table S10). Evidence of antileukemic activity, assessed by cumulative MMR rate, was observed independent of dose level (Fig. 3).

### Pharmacokinetics

As previously reported, PK assessments showed rapid absorption of asciminib in plasma, with concentrations peaking at 2–3 h after single or repeated oral dosing, regardless of dose level (Table S11) [30]. Based on these PK data and safety, tolerability, and preliminary efficacy results, asciminib 40 mg BID was chosen as the recommended dose for expansion in this patient population.

## DISCUSSION

This final analysis of the phase 1 study is the longest follow-up to date of asciminib monotherapy in patients with CML-CP without *BCR::ABL1*^T315I^ with ≤ 8.4 years of therapy. The results of this analysis are consistent with the previous analysis and demonstrated that long-term asciminib provides durable efficacy, is well tolerated, and has a favorable safety profile with no new safety signals. Overall, most AEs first occurred within the first year of treatment initiation, were grade 1/2, and were manageable with dose adjustments, dose interruption, or additional therapy. At the time of this final analysis, most AESIs had resolved or were resolving. No new on-treatment deaths occurred since the prior analysis [16]. This longer follow-up continued to show that the likelihood of a new AE does not increase with longer exposure to asciminib.

With increased exposure in this population, the most frequent AEs were largely consistent with those of previous analyses of asciminib, including the current study and ASCEMBL [15, 16, 25, 30]. The rate of grade ≥ 3 AEs in the final analysis and the 4-year follow-up were consistent, occurring in 88 and 83 patients, respectively [16]. After 5.9 years of median exposure, few patients (*n* = 15; 13.0%) discontinued treatment due to AEs. Together, these results demonstrated the long-term safety and tolerability of asciminib in heavily pretreated patients with CML-CP, a population including many who may have previously experienced resistance to and/or intolerance of TKIs and who have limited further treatment options [1, 10, 37].

Due to the possibility of lipase and amylase elevations occurring without symptoms of clinical pancreatitis, patients treated with TKIs should be monitored closely for clinical pancreatitis, and dose reduction must be applied if needed [8, 38, 39]. Since the previous analysis, no new patients experienced clinical pancreatitis [16]. Per study protocol, asymptomatic grade 3 or 4 lipase elevations or grade 2 pancreatitis (asymptomatic or symptomatic) that was not resolved with treatment interruption ≤ 21 days, or grade ≥ 3 pancreatitis required discontinuation of treatment. Pancreatic events leading to discontinuation included pancreatitis (*n* = 1), acute pancreatitis (*n* = 1), lipase increased (*n* = 4), and amylase increased (*n* = 2). As previously reported, among the dose-determining analysis set (*n* = 132 patients with CML-CP/AP), pancreatic events were a DLT of asciminib in 4 patients, including lipase increased (*n* = 2) and pancreatitis (*n* = 2, 2 episodes each) [16, 30]. In this analysis, most pancreatic events, including enzyme elevations, were resolved by the cutoff. With 2.3 years’ median follow-up in ASCEMBL, no cases of clinical pancreatitis occurred, and few patients who received asciminib experienced grade ≥3 lipase elevation (*n* = 6) and/or amylase elevation (*n* = 1) [15]. As with the present study, ASCEMBL excluded patients who had pancreatic events in the past 12 months or any history of chronic pancreatitis [40]. In preclinical studies, pancreatic toxicity was observed in dogs, but not in rats or monkeys [31]. However, the risk factors that contribute to the development and progression of pancreatic events, and the influence of TKIs and asciminib on pancreatic events, are unknown. Regular monitoring with dose modification as needed is recommended [26].

Most AOEs that occurred during the study were previously reported [16]. While no grade 4 AOEs occurred previously, 1 grade 4 AOE (cerebrovascular accident) occurred since the previous analysis [16]. Patients who experienced AOEs were heavily pretreated and/or had ≥1 past or active condition. Most ATP-competitive TKIs present a risk for AOEs, especially in patients with heavy TKI treatment history and/or comorbidities [5, 11, 15, 16, 41–44]. Therefore, patients should be closely monitored for the emergence of AOEs and comorbidities, especially if they have other risk factors [44]. In ASCEMBL, which assessed asciminib 40 mg BID, the risk of AOEs did not increase over time with a median exposure of 2.3 years [15]. Notably, asciminib displayed a consistent safety profile with previous analyses after ≤ 8.4 years’ exposure, with no new safety concerns.

In this final analysis of the phase 1 study, long-term efficacy results indicated durable antileukemic activity. MMR is a recognized treatment milestone associated with long-term survival [1]. In practice, MR is periodically monitored to make treatment decisions, such as the switching to a new TKI [1]. By this final analysis cutoff, MMR was achieved in 65.1% of patients.

While half of patients who achieved MMR did so by week 48 (year ≈ 1), cumulative MMR increased over time, and patients achieved first MMR as late as approximately year 7. This longer follow-up analysis demonstrates durable responses, with 50 of 56 patients who achieved MMR maintaining or deepening their response level.

In the previous analysis, 48 of 53 patients who achieved MMR maintained or achieved deeper responses by the cutoff date [16]. No obvious correlations were observed between MMR rate and treatment line, and clinically meaningful MMR rates were observed regardless of the number of prior TKIs or baseline *BCR::ABL1*^IS^ level [16]. Since the previous analysis, 1 additional patient who achieved MMR lost the response [16]. DMR is an important treatment goal [10], but many patients treated with ≥2 TKIs do not achieve DMR [11, 45], demonstrating a significant unmet need in this population.

In this final analysis, patients continued to achieve their first MMRs and DMRs even at later time points. MMR was durable over time with patients improving to deeper responses, demonstrating the potential for asciminib to improve outcomes in this heavily pretreated population for whom prolonged clinical benefit is difficult to sustain. By the time of the final cutoff, the median time to first MMR was 58.3 weeks (range, 2 to 360 weeks) or 1.1 years.

Patients with CML-CP without *BCR::ABL1*^T315I^ with multiple prior TKIs demonstrated reliable and durable MRs while using asciminib, with favorable safety and tolerability. These results, in addition to the results from ASCEMBL that showed superior efficacy with asciminib compared with bosutinib at weeks 24 (month ≈ 6) and 96 (year ≈ 2), support asciminib as a therapy of choice for patients with CML-CP after ≥ 2 TKIs without *BCR::ABL1*^T315I^ [15, 25].

From the dose-determining analysis set in the dose-escalation part of the study, DLTs occurred in 8 of 132 patients during the first cycle of treatment; as reported previously, a maximum tolerated dose was not reached [30]. In this patient population, 40 mg BID was the recommended phase 2 dose because it was observed to be safe and efficacious in the phase 1 trial and was predicted to provide asciminib blood concentrations above a preclinically defined inhibitory threshold in all patients with CML-CP without *BCR::ABL1*^T315I^ [16, 30, 46, 47]. With similar PK parameters and probabilities of DLTs between the QD and BID dosing regimens [16, 30, 31, 47], the 40-mg BID dose has been widely approved and the 80-mg QD dose has been approved in some countries for patients with CML-CP after ≥ 2 TKIs without *BCR::ABL1*^T315I^ [26, 27, 31].

Several ongoing studies are currently evaluating the safety and efficacy of the asciminib recommended doses of 40 mg BID and/or 80 mg QD as monotherapy or as combination therapy in patients with CML-CP who are newly diagnosed or previously treated with TKI(s) (AIM4CML [NCT04666259]; ASC4OPT [NCT04948333]; ASC4FIRST [NCT04971226]; ASC4START [NCT05456191]; ASC4MORE [NCT03578367]) [32, 48–50]. As the first and only approved BCR::ABL1 inhibitor that works by Specifically Targeting the ABL Myristoyl Pocket (STAMP) [24, 25, 28], asciminib is supported as a safe and tolerable long-term treatment with the potential to become the therapy of choice for patients with CML-CP.

## Supplementary Material

supplemental tables and figures

supplemental figure 1

supplemental figure 2

supplemental figure 3

**Supplementary information** The online version contains supplementary material available at https://doi.org/10.1038/s41375-025-02578-7.

## Figures and Tables

**Table 1.** Treatment-emergent adverse events (AEs; ≥ 10% of patients), regardless of relationship to study drug.

Category, n (%)^a^	All patients *N* = 115	
	All grades	Grade ≥ 3
**Number of patients with ≥ 1 event**	115 (100)	88 (76.5)

Arthralgia	47 (40.9)	3 (2.6)

Lipase increased^b^	45 (39.1)	25 (21.7)

Fatigue	44 (38.3)	2 (1.7)

Headache	44 (38.3)	3 (2.6)

Diarrhea	38 (33.0)	0

Vomiting	37 (32.2)	3 (2.6)

Nausea	36 (31.3)	2 (1.7)

Hypertension	35 (30.4)	21 (18.3)

Abdominal pain	32 (27.8)	1 (0.9)

Dizziness	31 (27.0)	0

Upper respiratory tract infection	31 (27.0)	1 (0.9)

COVID-19	29 (25.2)	3 (2.6)

Rash	29 (25.2)	0

Pruritus	27 (23.5)	1 (0.9)

Cough	26 (22.6)	0

Thrombocytopenia	26 (22.6)	12 (10.4)

Amylase increased	25 (21.7)	5 (4.3)

Myalgia	25 (21.7)	3 (2.6)

Constipation	24 (20.9)	1 (0.9)

Back pain	23 (20.0)	3 (2.6)

Pain in extremity	23 (20.0)	1 (0.9)

Upper abdominal pain	21 (18.3)	0

Anemia	19 (16.5)	10 (8.7)

Peripheral edema	19 (16.5)	0

Dyspnea	18 (15.7)	1 (0.9)

Increased ALT	17 (14.8)	3 (2.6)

Bone pain	17 (14.8)	2 (1.7)

Nasopharyngitis	17 (14.8)	0

Pyrexia	17 (14.8)	1 (0.9)

Increased weight	17 (14.8)	3 (2.6)

Neutropenia	16 (13.9)	10 (8.7)

Hyperhidrosis	15 (13.0)	0

Non-cardiac chest pain	15 (13.0)	0

Pleural effusion	15 (13.0)	4 (3.5)

Anxiety	14 (12.2)	1 (0.9)

Increased AST	14 (12.2)	0

Decreased appetite	14 (12.2)	0

Depression	14 (12.2)	0

Hypertriglyceridemia	14 (12.2)	3 (2.6)

Oropharyngeal pain	14 (12.2)	0

Increased blood creatinine	13 (11.3)	0

Hyperglycemia	13 (11.3)	2 (1.7)

Hyperuricemia	13 (11.3)	5 (4.3)

Insomnia	13 (11.3)	1 (0.9)

Dry eye	12 (10.4)	0

Fall	12 (10.4)	2 (1.7)

Increased γ-glutamyltransferase	12 (10.4)	3 (2.6)

Hypophosphatemia	12 (10.4)	3 (2.6)

Muscle spasms	12 (10.4)	0

Pneumonia	12 (10.4)	5 (4.3)

*ALT* alanine aminotransferase, *AST* aspartate aminotransferase.

a A patient with multiple grades of severity for an event was only counted under the maximum grade.

b Does not include the preferred term hyperlipasemia.

**Table 2.** Exposure-adjusted incidence rates (EAIR) of all-grade adverse events of special interest.

EAIR, *n* (per 100 patient treatment years)	All patients *N* = 115
Gastrointestinal events	85 (51.0)
Hypersensitivity	56 (17.1)
Pancreatic events (including isolated pancreatic enzyme elevations)	54 (15.7)
Pancreatitis (clinical events)^a^	8 (1.4)
Myelosuppression^b^	42 (9.4)
Thrombocytopenia	30 (6.1)
Anemia	21 (4.1)
Leukopenia	21 (4.1)
Neutropenia	19 (3.6)
Cytopenias affecting > 1 lineage	1 (0.2)
Hepatotoxicity (including laboratory terms)	36 (7.9)
Hepatotoxicity (clinical events)	5 (0.9)
Edema and fluid retention	35 (7.6)
Hemorrhage	27 (5.5)
Ischemic heart and CNS conditions	18 (3.5)
Ischemic heart disease	15 (2.8)
Ischemic CNS vascular conditions	5 (0.9)
Arterial occlusive events	14 (2.6)
Phototoxicity	11 (2.0)
Cardiac failure (clinical events)	9 (1.6)
QTc prolongation	9 (1.6)

*CNS* central nervous system, *QTc* corrected QT interval.

a Includes preferred terms pancreatitis and acute pancreatitis.

b Myelosuppression included erythropenia, leukopenia, thrombocytopenia, and cytopenias affecting more than one lineage.

## Data Availability

Novartis is committed to sharing with qualified external researchers, access to patient-level data and supporting clinical documents from eligible studies. These requests are reviewed and approved by an independent review panel on the basis of scientific merit. All data provided are anonymized to respect the privacy of patients who have participated in the trial in line with applicable laws and regulations. The trial data availability is according to the criteria and process described on http://www.clinicalstudydatarequest.com.
