# Supplementary material for: Asciminib monotherapy in patients with chronic myeloid leukemia in chronic phase without BCR::ABL1T315I treated with at least 2 prior TKIs: Phase 1 final results
Source: Leukemia. Author manuscript; Available in PMC 2026 May 1. (PMC12055594; doi:10.1038/s41375-025-02578-7)
Supplement: supplemental figure 1 [file NIHMS2076239-supplement-supplemental_figure_1.pdf]

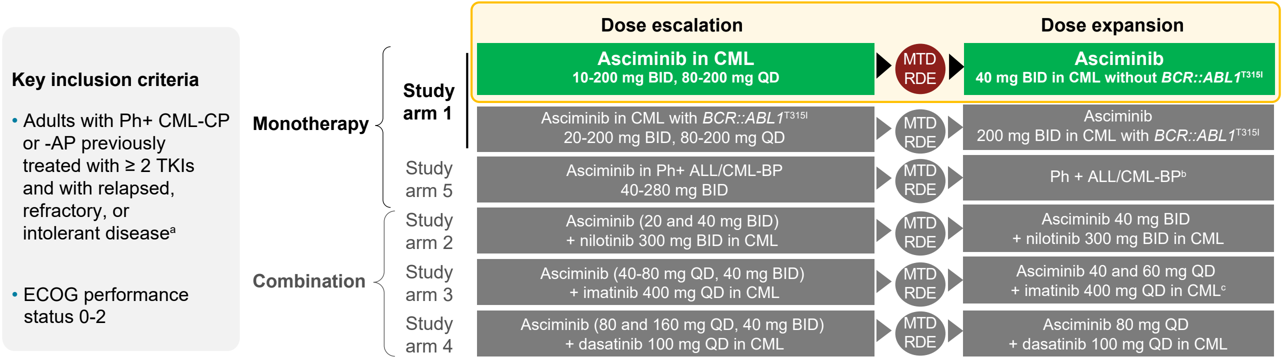
Key inclusion criteria
• Adults with Ph+ CML-CP or -AP previously treated with ≥ 2 TKIs and with relapsed, refractory, or intolerant disease[a]
• ECOG performance status 0-2
Monotherapy
Combination
Dose escalation
Dose expansion
Study arm 1
Asciminib in CML
10-200 mg BID, 80-200 mg QD
MTD RDE
Asciminib
40 mg BID in CML without BCR::ABL1 T315I
Asciminib in CML with BCR::ABL1 T315I
20-200 mg BID, 80-200 mg QD
MTD RDE
Asciminib
200 mg BID in CML with BCR::ABL1 T315I
Study arm 5
Asciminib in Ph+ ALL/CML-BP
40-280 mg BID
MTD RDE
Ph + ALL/CML-BP[b]
Study arm 2
Asciminib (20 and 40 mg BID)
+ nilotinib 300 mg BID in CML
MTD RDE
Asciminib 40 mg BID
+ nilotinib 300 mg BID in CML
Study arm 3
Asciminib (40-80 mg QD, 40 mg BID)
+ imatinib 400 mg QD in CML
MTD RDE
Asciminib 40 and 60 mg QD
+ imatinib 400 mg QD in CML[c]
Study arm 4
Asciminib (80 and 160 mg QD, 40 mg BID)
+ dasatinib 100 mg QD in CML
MTD RDE
Asciminib 80 mg QD
+ dasatinib 100 mg QD in CML
