# Supplementary material for: Asciminib monotherapy in patients with chronic myeloid leukemia in chronic phase without BCR::ABL1T315I treated with at least 2 prior TKIs: Phase 1 final results
Source: Leukemia. Author manuscript; Available in PMC 2026 May 1. (PMC12055594; doi:10.1038/s41375-025-02578-7)
Supplement: supplemental figure 2 [file NIHMS2076239-supplement-supplemental_figure_2.pdf]

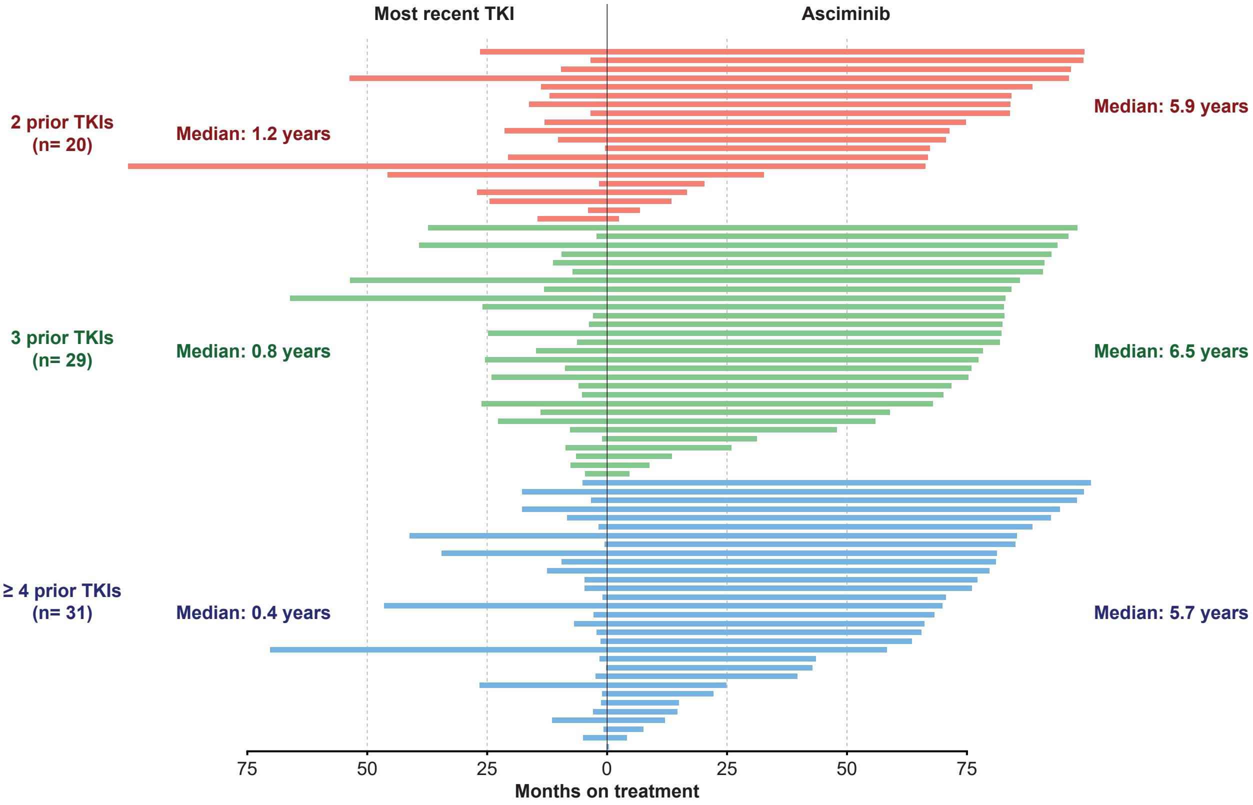
Most recent TKI
Asciminib
2 prior TKIs
(n= 20)
Median: 1.2 years
Median: 5.9 years
3 prior TKIs
(n= 29)
Median: 0.8 years
Median: 6.5 years
≥ 4 prior TKIs
(n= 31)
Median: 0.4 years
Median: 5.7 years
75
50
25
0
25
50
75
Months on treatment
