# Supplementary figures and images for: Asciminib monotherapy in patients with chronic myeloid leukemia in chronic phase without BCR::ABL1T315I treated with at least 2 prior TKIs: Phase 1 final results
Source: Leukemia. Author manuscript; Available in PMC 2026 May 1. (PMC12055594; doi:10.1038/s41375-025-02578-7)

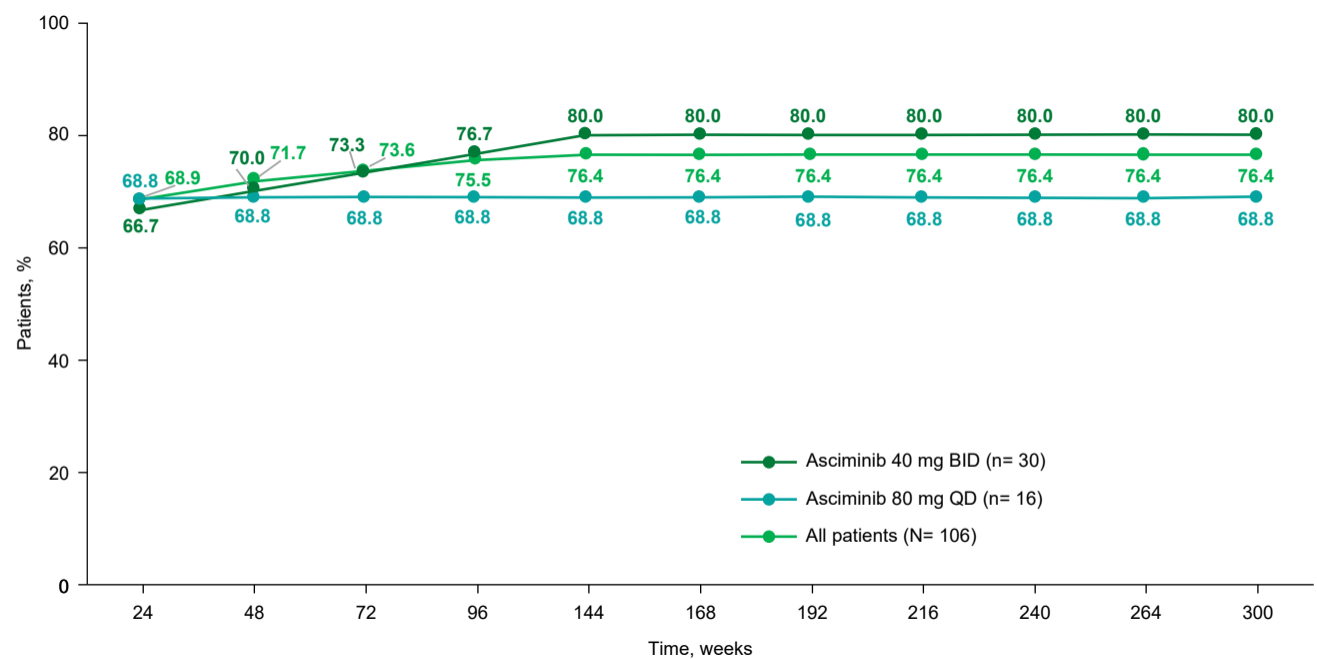

Supplement: supplemental figure 3 [file NIHMS2076239-supplement-supplemental_figure_3.pdf]
