# Supplementary material for: Asciminib monotherapy in patients with chronic myeloid leukemia in chronic phase without BCR::ABL1T315I treated with at least 2 prior TKIs: Phase 1 final results
Source: Leukemia. 2025 Apr 9;39(5):1114–23. doi: 10.1038/s41375-025-02578-7 (PMC12055594; doi:10.1038/s41375-025-02578-7)
Supplement: Supplementary file 1 [file 41375_2025_2578_MOESM1_ESM.docx]

**Supplemental Materials**

**Supplemental Tables and Figures**

Table S1. Number of patients receiving each starting dose of asciminib

| **Dose level, n** | **All patients** **N= 115** |
| --- | --- |
| **QD, mg** |  |
| 80 | 17 |
| 120 | 17 |
| 200 | 11 |
| **BID, mg** |  |
| 10 | 1 |
| 20 | 13 |
| 40 | 30 |
| 80 | 8 |
| 150 | 5 |
| 160 | 3 |
| 200 | 10 |

BID, twice daily; QD, once daily. 

Mauro MJ, et al. *Leukemia.* 2023;37,1048–1059.(1) Reprinted with permission.

Table S2. Patient demographics and clinical characteristics at baseline 

| **Variable** | **All patients (N= 115)** |
| --- | --- |
| **Age, median (range), years** | 56.0 (25-88) |
| **Age ≥ 65 years, n (%)** | 30 (26.1) |
| **Female, n (%)** | 55 (47.8) |
| **Race, n (%)** |  |
| Asian | 18 (15.7) |
| Black or African American | 3 (2.6) |
| Other | 10 (8.7) |
| Unknown | 2 (1.7) |
| White | 82 (71.3) |
| **Ethnicity, n (%)** |  |
| East Asian | 16 (13.9) |
| Hispanic or Latino | 7 (6.1) |
| Not reported | 22 (19.1) |
| Other | 57 (49.6) |
| Southeast Asian | 1 (0.9) |
| Unknown | 12 (10.4) |
| **ECOG performance status, n (%)** |  |
| 0 | 87 (75.7) |
| 1 | 26 (22.6) |
| 2 | 2 (1.7) |
| **Number of prior TKIs, n (%)** |  |
| 1 | 3 (2.6) |
| 2 | 30 (26.1) |
| 3 | 41 (35.7) |
| 4 | 32 (27.8) |
| ≥ 5 | 9 (7.8) |
| **Individual prior TKIs, n (%)** |  |
| Bosutinib | 45 (39.1) |
| Dasatinib | 98 (85.2) |
| Imatinib | 85 (73.9) |
| Nilotinib | 89 (77.4) |
| Ponatinib | 36 (31.3) |
| Radotinib | 6 (5.2) |
| Rebastinib | 1 (0.9) |
| **Molecular response at screening, n (%)** |  |
| *BCR::ABL1*^IS^ ≤ 0.0032% | 1 (0.9) |
| *BCR::ABL1*^IS^ > 0.0032 to ≤ 0.01% | 4 (3.5) |
| *BCR::ABL1*^IS^ > 0.01 to ≤ 0.1% | 15 (13.0) |
| *BCR::ABL1*^IS^ > 0.1 to ≤ 1% | 24 (20.9) |
| *BCR::ABL1*^IS^ > 1 to ≤ 10% | 21 (18.3) |
| *BCR::ABL1*^IS^ > 10% | 41 (35.7) |
| Atypical/e1a2/Unknown transcripts^a^ | 9 (7.8) |

ECOG, Eastern Cooperative Oncology Group; IS, International Scale; TKI, tyrosine kinase inhibitor.

^a^ Included *e1a2*, *e19a2*, *e1a3* minor transcripts, a novel variant, and unknown or not detected.

Mauro MJ, et al. *Leukemia.* 2023;37,1048–1059.(1) Reprinted with permission.

Table S3. Patient disposition by starting dose

| **Disposition, n (%)** | **Asciminib** **40 mg BID** **n= 30** | **Asciminib** **80 mg QD** **n= 17** | **All patients** **N= 115** |
| --- | --- | --- | --- |
| **Patients randomized** |  |  |  |
| Treated | 30 (100) | 17 (100) | 115 (100) |
| **Primary reason for discontinuation** |  |  |  |
| Adverse event | 5 (16.7) | 2 (11.8) | 15 (13.0) |
| Physician decision prior to the final analysis | 2 (6.7) | 2 (11.8) | 12 (10.4) |
| Lack of efficacy | 2 (6.7) | 2 (11.8) | 9 (7.8) |
| Other reasons^a^ | 0 | 0 | 3 (2.6) |
| Progressive disease | 4 (13.3) | 2 (11.8) | 8 (7.0) |
| Patient/guardian decision | 3 (10.0) | 1 (5.9) | 6 (5.2) |
| Death | 0 | 1 (5.9) | 2 (1.7) |
| Lost to follow-up | 1 (3.3) | 0 | 2 (1.7) |
| **Continued asciminib in post-trial access**^**b**^ | 15 (50) | 9 (52.9) | 70 (60.9) |

BID, twice daily; QD, once daily.

^a^ Other reasons included treatment-free remission attempt (n= 2), and other comorbidities (n= 1). 

^b^ Treatment ongoing as of the cutoff date (March 14, 2023) in post-trial access, including patients who continued to receive asciminib in a rollover study or via commercial availability.

Table S4. Treatment-emergent adverse events (≥ 10% of patients), regardless of relationship to study drug by starting dose

| **Category, n (%)** | **Asciminib** **40 mg BID** **n= 30** |  | **Asciminib** **80 mg QD** **n= 17** |  | **All patients** **N= 115** |  |
| --- | --- | --- | --- | --- | --- | --- |
|  | **All grades** | **Grade ≥ 3** | **All grades** | **Grade ≥ 3** | **All grades** | **Grade ≥ 3** |
| **Number of patients with ≥ 1 event** | 30 (100) | 24 (80.0) | 17 (100) | 15 (88.2) | 115 (100) | 88 (76.5) |
| Arthralgia | 12 (40.0) | 0 | 8 (47.1) | 2 (11.8) | 47 (40.9) | 3 (2.6) |
| Lipase increased^a^ | 16 (53.3) | 9 (30.0) | 4 (23.5) | 3 (17.6) | 45 (39.1) | 25 (21.7) |
| Fatigue | 13 (43.3) | 0 | 7 (41.2) | 0 | 44 (38.3) | 2 (1.7) |
| Headache | 9 (30.0) | 1 (3.3) | 7 (41.2) | 2 (11.8) | 44 (38.3) | 3 (2.6) |
| Diarrhea | 9 (30.0) | 0 | 5 (29.4) | 0 | 38 (33.0) | 0 |
| Vomiting | 11 (36.7) | 1 (3.3) | 3 (17.6) | 1 (5.9) | 37 (32.2) | 3 (2.6) |
| Nausea | 8 (26.7) | 0 | 5 (29.4) | 0 | 36 (31.3) | 2 (1.7) |
| Hypertension | 8 (26.7) | 4 (13.3) | 8 (47.1) | 6 (35.3) | 35 (30.4) | 21 (18.3) |
| Abdominal pain | 12 (40.0) | 0 | 5 (29.4) | 1 (5.9) | 32 (27.8) | 1 (0.9) |
| Dizziness | 6 (20.0) | 0 | 6 (35.3) | 0 | 31 (27.0) | 0 |
| Upper respiratory tract infection | 6 (20.0) | 0 | 8 (47.1) | 0 | 31 (27.0) | 1 (0.9) |
| COVID-19 | 7 (23.3) | 1 (3.3) | 5 (29.4) | 0 | 29 (25.2) | 3 (2.6) |
| Rash | 9 (30.0) | 0 | 4 (23.5) | 0 | 29 (25.2) | 0 |
| Pruritus | 5 (16.7) | 0 | 3 (17.6) | 0 | 27 (23.5) | 1 (0.9) |
| Cough | 8 (26.7) | 0 | 3 (17.6) | 0 | 26 (22.6) | 0 |
| Thrombocytopenia | 6 (20.0) | 2 (6.7) | 5 (29.4) | 2 (11.8) | 26 (22.6) | 12 (10.4) |
| Amylase increased | 7 (23.3) | 3 (10.0) | 3 (17.6) | 0 | 25 (21.7) | 5 (4.3) |
| Myalgia | 7 (23.3) | 2 (6.7) | 2 (11.8) | 0 | 25 (21.7) | 3 (2.6) |
| Constipation | 5 (16.7) | 0 | 4 (23.5) | 0 | 24 (20.9) | 1 (0.9) |
| Back pain | 5 (16.7) | 0 | 3 (17.6) | 2 (11.8) | 23 (20.0) | 3 (2.6) |
| Pain in extremity | 6 (20.0) | 0 | 2 (11.8) | 0 | 23 (20.0) | 1 (0.9) |
| Upper abdominal pain | 3 (10.0) | 0 | 3 (17.6) | 0 | 21 (18.3) | 0 |
| Anemia | 6 (20.0) | 4 (13.3) | 4 (23.5) | 0 | 19 (16.5) | 10 (8.7) |
| Peripheral edema | 6 (20.0) | 0 | 2 (11.8) | 0 | 19 (16.5) | 0 |
| Dyspnea | 5 (16.7) | 0 | 3 (17.6) | 0 | 18 (15.7) | 1 (0.9) |
| Increased ALT | 2 (6.7) | 0 | 2 (11.8) | 1 (5.9) | 17 (14.8) | 3 (2.6) |
| Bone pain | 5 (16.7) | 0 | 3 (17.6) | 1 (5.9) | 17 (14.8) | 2 (1.7) |
| Nasopharyngitis | 5 (16.7) | 0 | 0 | 0 | 17 (14.8) | 0 |
| Pyrexia | 5 (16.7) | 1 (3.3) | 1 (5.9) | 0 | 17 (14.8) | 1 (0.9) |
| Increased weight | 6 (20.0) | 1 (3.3) | 2 (11.8) | 0 | 17 (14.8) | 3 (2.6) |
| Neutropenia | 4 (13.3) | 3 (10.0) | 4 (23.5) | 2 (11.8) | 16 (13.9) | 10 (8.7) |
| Hyperhidrosis | 3 (10.0) | 0 | 3 (17.6) | 0 | 15 (13.0) | 0 |
| Noncardiac chest pain | 4 (13.3) | 0 | 2 (11.8) | 0 | 15 (13.0) | 0 |
| Pleural effusion | 1 (3.3) | 1 (3.3) | 1 (5.9) | 0 | 15 (13.0) | 4 (3.5) |
| Anxiety | 2 (6.7) | 0 | 2 (11.8) | 1 (5.9) | 14 (12.2) | 1 (0.9) |
| Increased AST | 2 (6.7) | 0 | 2 (11.8) | 0 | 14 (12.2) | 0 |
| Decreased appetite | 2 (6.7) | 0 | 0 | 0 | 14 (12.2) | 0 |
| Depression | 3 (10.0) | 0 | 1 (5.9) | 0 | 14 (12.2) | 0 |
| Hypertriglyceridemia | 4 (13.3) | 0 | 2 (11.8) | 1 (5.9) | 14 (12.2) | 3 (2.6) |
| Oropharyngeal pain | 3 (10.0) | 0 | 5 (29.4) | 0 | 14 (12.2) | 0 |
| Increased blood creatinine | 3 (10.0) | 0 | 1 (5.9) | 0 | 13 (11.3) | 0 |
| Hyperglycemia | 3 (10.0) | 0 | 2 (11.8) | 1 (5.9) | 13 (11.3) | 2 (1.7) |
| Hyperuricemia | 2 (6.7) | 2 (6.7) | 4 (23.5) | 2 (11.8) | 13 (11.3) | 5 (4.3) |
| Insomnia | 5 (16.7) | 0 | 1 (5.9) | 0 | 13 (11.3) | 1 (0.9) |
| Dry eye | 3 (10.0) | 0 | 1 (5.9) | 0 | 12 (10.4) | 0 |
| Fall | 3 (10.0) | 0 | 1 (5.9) | 0 | 12 (10.4) | 2 (1.7) |
| Increased γ-glutamyltransferase | 2 (6.7) | 0 | 1 (5.9) | 1 (5.9) | 12 (10.4) | 3 (2.6) |
| Hypophosphatemia | 4 (13.3) | 2 (6.7) | 1 (5.9) | 1 (5.9) | 12 (10.4) | 3 (2.6) |
| Muscle spasms | 5 (16.7) | 0 | 1 (5.9) | 0 | 12 (10.4) | 0 |
| Pneumonia | 3 (10.0) | 2 (6.7) | 2 (11.8) | 1 (5.9) | 12 (10.4) | 5 (4.3) |

ALT, alanine aminotransferase; AST, aspartate aminotransferase; BID, twice daily; QD, once daily.

^a^ Does not include the preferred term hyperlipasemia. 

Table S5. Adverse events (AEs) leading to treatment discontinuation by starting dose

| **Category, n (%)**^**a**^ | **Asciminib** **40 mg BID** **n= 30** | **Asciminib** **80 mg QD** **n= 17** | **All patients** **N= 115** |
| --- | --- | --- | --- |
|  | **All grades** | **All grades** | **All grades** |
| **Number of patients with ≥ 1 event** | 4 (13.3) | 3 (17.6) | 15 (13.0) |
| Increased lipase^b^ | 1 (3.3) | 0 | 4 (3.5) |
| Increased amylase^c^ | 1 (3.3) | 0 | 2 (1.7) |
| Thrombocytopenia^d^ | 0 | 0 | 3 (2.6) |
| Pancreatitis^e^ | 2 (6.7) | 0 | 2 (1.7) |
| Acute kidney injury^f^ | 0 | 1 (5.9) | 1 (0.9) |
| Bronchospasm^g^ | 0 | 0 | 1 (0.9) |
| Cardiac arrest^f^ | 0 | 1 (5.9) | 1 (0.9) |
| Cyanosis^g^ | 0 | 0 | 1 (0.9) |
| General physical health deterioration | 0 | 0 | 1 (0.9) |
| Leukocytosis | 0 | 1 (5.9) | 1 (0.9) |
| Myalgia^h^ | 1 (3.3) | 0 | 1 (0.9) |
| Neutropenia^h,i^ | 0 | 0 | 1 (0.9) |
| Rash^g^ | 0 | 0 | 1 (0.9) |
| Thrombocytosis | 0 | 1 (5.9) | 1 (0.9) |

BID, twice daily; QD, once daily.

^a^ Patients with multiple grades of severity for an AE were only counted under the maximum grade.

^b^ Does not include the preferred term hyperlipasemia

^c^ The 2 patients who discontinued due to increased amylase also discontinued due to increased lipase.

^d ^Includes thrombocytopenia and platelet count decreased. 

^e^ Includes pancreatitis and pancreatitis acute. 

f Occurred in the same patient.

^g^ Occurred in the same patient.

^h^ New AEs leading to discontinuation since the previous analysis; each occurred in different patients. 

^i^ The patient who discontinued due to neutropenia also discontinued due to thrombocytopenia. 

Table S6. Adverse events (AEs) leading to study treatment dose adjustment or interruption, suspected to be related to study treatment

| **Category, n (%)**^**a**^ | **Asciminib** **40 mg BID** **n= 30** | **Asciminib** **80 mg QD** **n= 17** | **All patients** **N= 115** |
| --- | --- | --- | --- |
|  | **All grades** | **All grades** | **All grades** |
| **Patients with ≥ 1 AE requiring dose adjustment or interruption** | 13 (43.3) | 8 (47.1) | 51 (44.3) |
| Lipase increased | 6 (20.0) | 2 (11.8) | 20 (17.4) |
| Thrombocytopenia | 0 | 2 (11.8) | 8 (7.0) |
| Amylase increased | 2 (6.7) | 1 (5.9) | 6 (5.2) |
| Pancreatitis | 0 | 1 (5.9) | 5 (4.3) |
| Abdominal pain | 0 | 1 (5.9) | 4 (3.5) |
| Pleural effusion | 0 | 0 | 4 (3.5) |
| Neutropenia | 1 (3.3) | 1 (5.9) | 3 (2.6) |
| Fatigue | 1 (3.3) | 0 | 3 (2.6) |
| Platelet count decreased | 1 (3.3) | 1 (5.9) | 3 (2.6) |
| Neutrophil count decreased | 0 | 1 (5.9) | 2 (1.7) |
| Nausea | 2 (6.7) | 0 | 2 (1.7) |
| Hyperlipasemia | 2 (6.7) | 0 | 2 (1.7) |
| Arthralgia | 1 (3.3) | 0 | 2 (1.7) |
| Myalgia | 1 (3.3) | 0 | 2 (1.7) |
| Rash | 0 | 1 (5.9) | 2 (1.7) |
| Anemia | 0 | 0 | 1 (0.9) |
| Febrile neutropenia | 0 | 0 | 1 (0.9) |
| Leukopenia | 0 | 1 (5.9) | 1 (0.9) |
| Myelosuppression | 0 | 0 | 1 (0.9) |
| Vertigo | 0 | 0 | 1 (0.9) |
| Abdominal discomfort | 0 | 0 | 1 (0.9) |
| Pancreatitis acute | 0 | 0 | 1 (0.9) |
| Pancreatitis chronic | 0 | 0 | 1 (0.9) |
| Vomiting | 0 | 0 | 1 (0.9) |
| Feeling abnormal | 0 | 0 | 1 (0.9) |
| Noncardiac chest pain | 0 | 0 | 1 (0.9) |
| Tendon rupture | 0 | 1 (5.9) | 1 (0.9) |
| Blood creatinine increased | 1 (3.3) | 0 | 1 (0.9) |
| Gamma-glutamyltransferase increased | 0 | 1 (5.9) | 1 (0.9) |
| White blood cell count decreased | 0 | 1 (5.9) | 1 (0.9) |
| Hyperglycemia | 0 | 0 | 1 (0.9) |
| Hypertriglyceridemia | 0 | 1 (5.9) | 1 (0.9) |
| Back pain | 0 | 0 | 1 (0.9) |
| Flank pain | 0 | 0 | 1 (0.9) |
| Musculoskeletal chest pain | 0 | 0 | 1 (0.9) |
| Tendon disorder | 0 | 1 (5.9) | 1 (0.9) |
| Headache | 0 | 0 | 1 (0.9) |
| Insomnia | 0 | 0 | 1 (0.9) |
| Dyspnea | 1 (3.3) | 0 | 1 (0.9) |
| Pleurisy | 0 | 0 | 1 (0.9) |
| Dermatitis bullous | 0 | 0 | 1 (0.9) |
| Erythema | 0 | 0 | 1 (0.9) |
| Pruritis | 0 | 0 | 1 (0.9) |
| Skin ulcer | 0 | 0 | 1 (0.9) |
| Hypertension | 0 | 0 | 1 (0.9) |
| Rash pruritic | 0 | 0 | 1 (0.9) |
| Angina pectoris | 0 | 0 | 1 (0.9) |
| Cardiac failure | 0 | 0 | 1 (0.9) |
| Myocardial infarction | 0 | 0 | 1 (0.9) |
| Pericardial effusion | 0 | 0 | 1 (0.9) |

Table S7. Adverse events of special interest by starting dose

| **Patients, n (%)**^**a**^ | **Asciminib** **40 mg BID** **n= 30** |  | **Asciminib** **80 mg QD** **n= 17** |  | **All patients** **N= 115** |  |
| --- | --- | --- | --- | --- | --- | --- |
|  | **All grades** | **Grade ≥ 3** | **All grades** | **Grade ≥ 3** | **All grades** | **Grade ≥ 3** |
| GI events | 22 (73.3) | 1 (3.3) | 10 (58.8) | 2 (11.8) | 85 (73.9) | 6 (5.2) |
| Hypersensitivity^b^ | 14 (46.7) | 1 (3.3) | 8 (47.1) | 1 (5.9) | 56 (48.7) | 4 (3.5) |
| Pancreatic events (including isolated pancreatic enzyme elevations) | 17 (56.7) | 11 (36.7) | 6 (35.3) | 3 (17.6) | 54 (47.0) | 32 (27.8) |
| Pancreatitis (clinical events)^c^ | 3 (10.0) | 1 (3.3) | 1 (5.9) | 0 | 8 (7.0) | 4 (3.5) |
| Myelosuppression^d^ | 12 (40.0) | 7 (23.3) | 8 (47.1) | 3 (17.6) | 42 (36.5) | 23 (20.0) |
| Thrombocytopenia | 9 (30.0) | 5 (16.7) | 5 (29.4) | 2 (11.8) | 30 (26.1) | 16 (13.9) |
| Anemia | 6 (20.0) | 4 (13.3) | 5 (29.4) | 0 | 21 (18.3) | 10 (8.7) |
| Leukopenia | 5 (16.7) | 5 (16.7) | 5 (29.4) | 3 (17.6) | 21 (18.3) | 16 (13.9) |
| Neutropenia | 5 (16.7) | 5 (16.7) | 5 (29.4) | 3 (17.6) | 19 (16.5) | 14 (12.2) |
| Cytopenias affecting > 1 lineage | 0 | 0 | 0 | 0 | 1 (0.9) | 0 |
| Hepatic events (including elevations of aminotransferases and/or bilirubin) | 9 (30.0) | 0 | 4 (23.5) | 1 (5.9) | 36 (31.3) | 6 (5.2) |
| Hepatotoxicity (clinical events) | 2 (6.7) | 0 | 1 (5.9) | 0 | 5 (4.3) | 1 (0.9) |
| Edema and fluid retention | 7 (23.3) | 2 (6.7) | 4 (23.5) | 0 | 35 (30.4) | 5 (4.3) |
| Hemorrhage | 7 (23.3) | 1 (3.3) | 3 (17.6) | 0 | 27 (23.5) | 5 (4.3) |
| Ischemic heart and CNS conditions | 1 (3.3) | 0 | 4 (23.5) | 3 (17.6) | 18 (15.7) | 8 (7.0) |
| Ischemic heart disease | 1 (3.3) | 0 | 4 (23.5) | 2 (11.8) | 15 (13.0) | 6 (5.2) |
| Ischemic CNS vascular conditions | 0 | 0 | 1 (5.9) | 1 (5.9) | 5 (4.3) | 2 (1.7) |
| Arterial occlusive events^e^ | 1 (3.3) | 0 | 4 (23.5) | 3 (17.6) | 14 (12.2) | 7 (6.1) |
| Cardiac events | 1 (3.3) | 0 | 4 (23.5) | 2 (11.8) | 10 (8.7) | 4 (3.5) |
| Angina pectoris^f,g,h^ | 1 (3.3) | 0 | 2 (11.8) | 0 | 7 (6.1) | 0 |
| Myocardial infarction^g,h^ | 0 | 0 | 0 | 0 | 2 (1.7) | 2 (1.7) |
| Myocardial ischemia^h^ | 0 | 0 | 1 (5.9) | 1 (5.9) | 2 (1.7) | 1 (0.9) |
| Coronary artery disease | 0 | 0 | 1 (5.9) | 1 (5.9) | 1 (0.9) | 1 (0.9) |
| Troponin T increased^f^ | 0 | 0 | 1 (5.9) | 0 | 1 (0.9) | 0 |
| Cerebrovascular events | 0 | 0 | 1 (5.9) | 1 (5.9) | 6 (5.2) | 2 (1.7) |
| Carotid artery stenosis | 0 | 0 | 0 | 0 | 2 (1.7) | 1 (0.9) |
| Cerebellar infarction | 0 | 0 | 0 | 0 | 1 (0.9) | 0 |
| Cerebrovascular accident^f^ | 0 | 0 | 1 (5.9) | 1 (5.9) | 2 (1.7) | 1 (0.9) |
| Amaurosis fugax | 0 | 0 | 0 | 0 | 1 (0.9) | 0 |
| Peripheral events | 0 | 0 | 0 | 0 | 1 (0.9) | 1 (0.9) |
| Peripheral arterial occlusive disease^i^ | 0 | 0 | 0 | 0 | 1 (0.9) | 1 (0.9) |
| Phototoxicity | 2 (6.7) | 0 | 2 (11.8) | 0 | 11 (9.6) | 0 |
| Cardiac failure (clinical events) | 3 (10.0) | 1 (3.3) | 3 (17.6) | 3 (17.6) | 9 (7.8) | 6 (5.2) |
| QTc prolongation | 1 (3.3) | 0 | 5 (29.4) | 2 (11.8) | 9 (7.8) | 3 (2.6) |

AE, adverse event; BID, twice daily; CNS, central nervous system; GI, gastrointestinal; QD, once daily; QTc, correct QT interval.

^a^ A subject with multiple severity grades for an AE was only counted under the maximum grade. 

^b^ Included preferred terms allergic conjunctivitis, periorbital edema, swollen tongue, lip swelling, face edema, face swelling, drug hypersensitivity, allergic rhinitis, bronchospasm, rash, pustular rash, maculopapular rash, follicular rash, pruritic rash, erythematous rash, macular rash, dermatitis, bullous dermatitis, allergic dermatitis, atopic dermatitis, acneiform dermatitis, eczema, urticaria, contrast media reaction, and circulatory collapse. 

^c^ Includes preferred terms pancreatitis and acute pancreatitis. 

^d^ Myelosuppression included erythropenia, leukopenia, thrombocytopenia, and cytopenias affecting more than one lineage. 

^e^ Arterial occlusive events included the following *Medical Dictionary for Regulatory Activities* terms: embolic and thrombotic events, arterial; ischemic CNS vascular conditions; ischemic heart disease; myocardial infarction.

^f^ Angina pectoris (grade 2), troponin T increased (grade 1), and cerebrovascular accident (grade 4) were reported in the same patient.

^g^ In 1 patient, angina pectoris (grade 2) and myocardial infarction (grade 3) were reported.

^h^ In 1 patient, angina pectoris (grade 2), myocardial infarction (grade 3), and myocardial ischemia (grade 2) were reported.

^i^ The patient with peripheral arterial occlusive disease (grade 3) also experienced arterial bypass occlusion (grade 3). 

Table S8. Patients with arterial occlusive events since the previous cutoff

| **Asciminib starting dose** | **Age, years** | **Sex** | **Preferred term** | **Study day of occurrence** | **Baseline CV risk factors and relevant medical history** | **Prior TKIs** |
| --- | --- | --- | --- | --- | --- | --- |
| 20 mg BID | 55 | F | Angina pectoris (grade 2) | 1756 | No relevant medical history Active arthralgia, hypertension, muscle spasms, myalgia, chronic kidney disease, hyperuricemia, fatigue, constipation, night sweats, anxiety, rash, and increased blood creatinine | Nilotinib, imatinib, and dasatinib |
| 40 mg BID | 58 | F | Angina pectoris (grade 1) | 266 | Previous increased lipase Active type II diabetes mellitus, hypothyroidism, hypertension, dyspnea, gout, fatigue, hyperlipidemia, renal failure, pleural effusion, skin lesion, hyperuricemia, vitamin D deficiency, muscle spasms, nausea, cough, pain, osteoarthritis, hypomagnesemia, increased blood creatinine and chronic kidney disease | Nilotinib, imatinib, dasatinib |
| 20 mg BID | 65 | F | Amaurosis fugax (grade 2) Carotid artery stenosis (grade 3) | 1964 2011 | No relevant medical history Active hypertension | Nilotinib, dasatinib, and ponatinib |
| 80 mg QD^a^ | 59 | F | Angina pectoris (grade 2) Troponin T increased (grade 1) Cerebrovascular accident (grade 4) | 1857 1857 2063 | Previous visual impairment, dyspnea, and deep vein thrombosis Active obesity, seasonal allergy, tumor lysis syndrome, nephrolithiasis, cardiomyopathy, fatigue, cough, fibromyalgia, hypertrophic cardiomyopathy, essential hypertension, goiter, bronchial hyper reactivity, blurred vision, pain, asthma, chronic bronchitis, gastroesophageal reflux disease, irritable bowel syndrome, hyperlipidemia, and arthralgia | Imatinib, nilotinib, dasatinib, and bosutinib |

BID, twice daily;CV, cardiovascular; QD, once daily; TKI, tyrosine kinase inhibitor.

^a^ This patient also experienced two episodes of grade 3 cardiac failure congestive and two episodes of grade 3 atrial fibrillation.

Table S9. Molecular responses in evaluable patients at time points by starting dose^a^

|  | **Asciminib** **40 mg BID** **n= 30** | **Asciminib** **80 mg QD** **n= 16** | **All patients** **N= 106** |
| --- | --- | --- | --- |
| ***BCR::ABL1***^**IS**^ **> 10%, n (%)** |  |  |  |
| Week 24 (≈  month 6) | 3 (10.0) | 3 (18.8) | 14 (13.2) |
| Week 48 (≈  year 1) | 1 (3.3) | 3 (18.8) | 7 (6.6) |
| Week 96 (≈  year 2) | 1 (3.3) | 3 (18.8) | 7 (6.6) |
| Week 144 (≈  year 3) | 1 (3.3) | 1 (6.3) | 6 (5.7) |
| Week 192 (≈  year 4) | 0 | 0 | 3 (2.8) |
| Week 264 (≈  year 5) | 0 | 1 (6.3) | 4 (3.8) |
| Week 312 (≈  year 6) | 0 | 0 | 2 (1.9) |
| Week 360 (≈  year 7) | 0 | 0 | 1 (0.9) |
| Week 408 (≈  year 8) | 0 | 0 | 0 |
| **MR**^**1**^**, n (%)** |  |  |  |
| Week 24 (≈  month 6) | 25 (83.3) | 12 (75.0) | 82 (77.4) |
| Week 48 (≈  year 1) | 20 (66.7) | 12 (75.0) | 80 (75.5) |
| Week 96 (≈  year 2) | 18 (60.0) | 11 (68.8) | 74 (69.8) |
| Week 144 (≈  year 3) | 19 (63.3) | 10 (62.5) | 69 (65.1) |
| Week 192 (≈  year 4) | 19 (63.3) | 7 (43.8) | 66 (62.3) |
| Week 264 (≈  year 5) | 14 (46.7) | 7 (43.8) | 54 (50.9) |
| Week 312 (≈  year 6) | 14 (46.7) | 7 (43.8) | 50 (47.2) |
| Week 360 (≈  year 7) | 12 (40.0) | 0 | 35 (33.0) |
| Week 408 (≈  year 8) | 3 (10.0) | 0 | 15 (14.2) |
| **MMR, n (%)** |  |  |  |
| Week 24 (≈  month 6) | 7 (23.3) | 6 (37.5) | 35 (33.0) |
| Week 48 (≈  year 1) | 5 (16.7) | 7 (43.8) | 39 (36.8) |
| Week 96 (≈  year 2) | 11 (36.7) | 8 (50.0) | 50 (47.2) |
| Week 144 (≈  year 3) | 13 (43.3) | 8 (50.0) | 56 (52.8) |
| Week 192 (≈  year 4) | 15 (50.0) | 5 (31.3) | 55 (51.9) |
| Week 264 (≈  year 5) | 8 (26.7) | 6 (37.5) | 44 (41.5) |
| Week 312 (≈  year 6) | 11 (36.7) | 6 (37.5) | 41 (38.7) |
| Week 360 (≈  year 7) | 11 (36.7) | 0 | 31 (29.2) |
| Week 408 (≈  year 8) | 3 (10.0) | 0 | 12 (11.3) |
| **MR**^**4**^**, n (%)** |  |  |  |
| Week 24 (≈  month 6) | 5 (16.7) | 3 (18.8) | 20 (18.9) |
| Week 48 (≈  year 1) | 4 (13.3) | 2 (12.5) | 19 (17.9) |
| Week 96 (≈  year 2) | 4 (13.3) | 6 (37.5) | 25 (23.6) |
| Week 144 (≈  year 3) | 5 (16.7) | 5 (31.3) | 29 (27.4) |
| Week 192 (≈  year 4) | 4 (13.3) | 3 (18.8) | 24 (22.6) |
| Week 264 (≈  year 5) | 2 (6.7) | 3 (18.8) | 20 (18.9) |
| Week 312 (≈  year 6) | 3 (10.0) | 4 (25.0) | 21 (19.8) |
| Week 360 (≈  year 7) | 5 (16.7) | 0 | 15 (14.2) |
| Week 408 (≈  year 8) | 1 (3.3) | 0 | 8 (7.5) |
| **MR**^**4.5**^**, n (%)** |  |  |  |
| Week 24 (≈  month 6) | 4 (13.3) | 2 (12.5) | 14 (13.2) |
| Week 48 (≈  year 1) | 3 (10.0) | 2 (12.5) | 16 (15.1) |
| Week 96 (≈  year 2) | 4 (13.3) | 3 (18.8) | 20 (18.9) |
| Week 144 (≈  year 3) | 4 (13.3) | 5 (31.3) | 23 (21.7) |
| Week 192 (≈  year 4) | 2 (6.7) | 2 (12.5) | 19 (17.9) |
| Week 264 (≈  year 5) | 2 (6.7) | 2 (12.5) | 16 (15.1) |
| Week 312 (≈  year 6) | 1 (3.3) | 3 (18.8) | 14 (13.2) |
| Week 360 (≈  year 7) | 3 (10.0) | 0 | 8 (7.5) |
| Week 408 (≈  year 8) | 0 | 0 | 4 (3.8) |
| **Missing** |  |  |  |
| Week 24 (≈  month 6) | 2 (6.7) | 1 (6.3) | 10 (9.4) |
| Week 48 (≈  year 1) | 9 (30.0) | 1 (6.3) | 19 (17.9) |
| Week 96 (≈  year 2) | 11 (36.7) | 2 (12.5) | 25 (23.6) |
| Week 144 (≈  year 3) | 10 (33.3) | 5 (31.3) | 31 (29.2) |
| Week 192 (≈  year 4) | 11 (36.7) | 9 (56.3) | 37 (34.9) |
| Week 264 (≈  year 5) | 16 (53.3) | 8 (50.0) | 48 (45.3) |
| Week 312 (≈  year 6) | 16 (53.3) | 9 (56.3) | 54 (50.9) |
| Week 360 (≈  year 7) | 18 (60.0) | 16 (100.0) | 70 (66.0) |
| Week 408 (≈  year 8) | 27 (90.0) | 16 (100.0) | 91 (85.8) |

BID, twice daily; DMR, deep molecular response; IS, International Scale; MMR, major molecular response (*BCR::ABL1*^IS^ ≤ 0.1%); MR^1^, *BCR::ABL1*^IS^ ≤ 10%;  MR^4^, *BCR::ABL1*^IS^ ≤ 0.01%; MR^4.5^, *BCR::ABL1*^IS^ ≤ 0.0032%; QD, once daily.

^**a**^ Included all patients with evaluable transcripts not expressing atypical, p190, or unknown transcripts and not having a missing evaluation at screening.

Table S10. Cumulative molecular responses in evaluable patients by time points by starting dose^a^

|  | **Asciminib** **40 mg BID** **n= 30** | **Asciminib** **80 mg QD** **n= 16** | **All patients** **N= 106** |
| --- | --- | --- | --- |
| ***BCR::ABL1***^**IS**^ **> 10%, n (%)** |  |  |  |
| Week 24 (≈  month 6) | 9 (30.0) | 6 (37.5) | 35 (33.0) |
| Week 48 (≈  year 1) | 9 (30.0) | 6 (37.5) | 35 (33.0) |
| Week 96 (≈  year 2) | 9 (30.0) | 6 (37.5) | 36 (34.0) |
| Week 144 (≈  year 3) | 9 (30.0) | 6 (37.5) | 37 (34.9) |
| Week 192 (≈ year 4) | 9 (30.0) | 6 (37.5) | 37 (34.9) |
| Week 264 (≈ year 5) | 9 (30.0) | 7 (43.8) | 38 (35.8) |
| Week 300 (year 5.7) | 10 (33.3) | 7 (43.8) | 39 (36.8) |
| ***BCR::ABL1***^**IS**^ **≤ 10%, n (%)** |  |  |  |
| Week 24 (≈ month 6) | 27 (90.0) | 12 (75.0) | 85 (80.2) |
| Week 48 (≈ year 1) | 27 (90.0) | 12 (75.0) | 88 (83.0) |
| Week 96 (≈ year 2) | 27 (90.0) | 12 (75.0) | 88 (83.0) |
| Week 144 (≈ year 3) | 27 (90.0) | 12 (75.0) | 88 (83.0) |
| Week 192 (≈ year 4) | 27 (90.0) | 12 (75.0) | 88 (83.0) |
| Week 264 (≈ year 5) | 27 (90.0) | 12 (75.0) | 88 (83.0) |
| Week 300 (year 5.7) | 27 (90.0) | 12 (75.0) | 88 (83.0) |
| ***BCR::ABL1***^**IS**^ **≤ 1%, n (%)** |  |  |  |
| Week 24 (≈ month 6) | 20 (66.7) | 11 (68.8) | 73 (68.9) |
| Week 48 (≈ year 1) | 21 (70.0) | 11 (68.8) | 76 (71.7) |
| Week 96 (≈ year 2) | 23 (76.7) | 11 (68.8) | 80 (75.5) |
| Week 144 (≈ year 3) | 24 (80.0) | 11 (68.8) | 81 (76.4) |
| Week 192 (≈ year 4) | 24 (80.0) | 11 (68.8) | 81 (76.4) |
| Week 264 (≈ year 5) | 24 (80.0) | 11 (68.8) | 81 (76.4) |
| Week 300 (year 5.7) | 24 (80.0) | 11 (68.8) | 81 (76.4) |
| **MMR, n (%)** |  |  |  |
| Week 24 (≈ month 6) | 7 (23.3) | 6 (37.5) | 37 (34.9) |
| Week 48 (≈ year 1) | 10 (33.3) | 7 (43.8) | 46 (43.4) |
| Week 96 (≈ year 2) | 15 (50.0) | 8 (50.0) | 55 (51.9) |
| Week 144 (≈ year 3) | 18 (60.0) | 9 (56.3) | 66 (62.3) |
| Week 192 (≈ year 4) | 19 (63.3) | 9 (56.3) | 69 (65.1) |
| Week 264 (≈ year 5) | 20 (66.7) | 10 (62.5) | 71 (67.0) |
| Week 300 (year 5.7) | 21 (70.0) | 10 (62.5) | 72 (67.9) |
| **MR**^**4**^**, n (%)** |  |  |  |
| Week 24 (≈ month 6) | 5 (16.7) | 3 (18.8) | 20 (18.9) |
| Week 48 (≈ year 1) | 6 (20.0) | 4 (25.0) | 24 (22.6) |
| Week 96 (≈ year 2) | 6 (20.0) | 6 (37.5) | 28 (26.4) |
| Week 144 (≈ year 3) | 7 (23.3) | 6 (37.5) | 32 (30.2) |
| Week 192 (≈ year 4) | 7 (23.3) | 6 (37.5) | 34 (32.1) |
| Week 264 (≈ year 5) | 8 (26.7) | 7 (43.8) | 39 (36.8) |
| Week 300 (year 5.7) | 9 (30.0) | 7 (43.8) | 42 (39.6) |
| **MR**^**4.5**^**, n (%)** |  |  |  |
| Week 24 (≈ month 6) | 5 (16.7) | 2 (12.5) | 15 (14.2) |
| Week 48 (≈ year 1) | 6 (20.0) | 2 (12.5) | 20 (18.9) |
| Week 96 (≈ year 2) | 6 (20.0) | 3 (18.8) | 23 (21.7) |
| Week 144 (≈ year 3) | 6 (20.0) | 5 (31.3) | 26 (24.5) |
| Week 192 (≈ year 4) | 6 (20.0) | 5 (31.3) | 28 (26.4) |
| Week 264 (≈ year 5) | 7 (23.3) | 6 (37.5) | 32 (30.2) |
| Week 300 (year 5.7) | 7 (23.3) | 6 (37.5) | 33 (31.1) |
| **Missing** |  |  |  |
| Week 24 (≈ month 6) | 23 (76.7) | 15 (93.8) | 86 (81.1) |
| Week 48 (≈ year 1) | 26 (86.7) | 15 (93.8) | 90 (84.9) |
| Week 96 (≈ year 2) | 28 (93.3) | 15 (93.8) | 93 (87.7) |
| Week 144 (≈ year 3) | 28 (93.3) | 15 (93.8) | 94 (88.7) |
| Week 192 (≈ year 4) | 29 (96.7) | 15 (93.8) | 97 (91.5) |
| Week 264 (≈ year 5) | 29 (96.7) | 15 (93.8) | 103 (97.2) |
| Week 300 (year 5.7) | 29 (96.7) | 15 (98.3) | 104 (98.1) |

BID, twice daily; DMR, deep molecular response; IS, International Scale; MMR, major molecular response (*BCR::ABL1*^IS^ ≤ 0.1%); MR^1^, *BCR::ABL1*^IS^ ≤ 10%;  MR^4^, *BCR::ABL1*^IS^ ≤ 0.01%; MR^4.5^, *BCR::ABL1*^IS^ ≤ 0.0032%; QD, once daily.

^a^ Included all patients with evaluable transcripts not expressing an atypical, p190, or unknown transcripts and not having a missing evaluation at screening.

Table S11. Pharmacokinetics parameters of asciminib following oral administration: single dose and steady state^a^

| **Cycle** | **Day** | **Treatment group** | **n** | **T**_**max**_**,** **, median (range), hr** | **C**_**max**_**,** **, geometric mean (CV%), ng/mL** | **AUC**_**last**_**, , geometric mean (CV%), hr•ng/mL**^**b**^ | **C**_**trough**_ **(predose visit),  geometric mean (CV%), ng/mL** |
| --- | --- | --- | --- | --- | --- | --- | --- |
| 1 | 1 | 10 mg BID (N= 1) | 1 | 3.0 | 123.0 | 533.83 | NR |
|  |  | 20 mg BID (N= 14) | 14 | 2.07 (1.83-3.10) | 233.26 (39.07) | 989.79 (40.96) | NR |
|  |  | 40 mg BID (N= 32) | 30 | 2.10 (1.95-5.62) | 536.85 (74.29) | 2247.11 (69.26) | NR |
|  |  | 80 mg BID (N= 12) | 12 | 2.88 (1.00-3.93) | 1284.29 (36.98) | 5457.68 (37.08) | NR |
|  |  | 150 mg BID N= 13) | 13 | 2.03 (1.92-4.00) | 2278.03 (50.84) | 10413.5 (43.05) | NR |
|  |  | 160 mg BID (N= 11) | 11 | 2.10 (0.83-5.98) | 2525.86 (65.54) | 9835.66 (64.99) | NR |
|  |  | 200 mg BID (N= 62) | 61 | 2.03 (0.95-7.28) | 3464.70 (33.60) | 14869.9 (33.42) | NR |
|  |  | 80 mg QD (N= 18) | 18 | 2.06 (1.13-6.00) | 1157.54 (46.46) | 10752.4 (42.90) | NR |
|  |  | 120 mg QD (N= 22) | 22 | 2.04 (1.13-7.65) | 2118.57 (28.79) | 16874.1 (31.47) | NR |
|  |  | 200 mg QD (N= 12) | 12 | 2.00 (1.08-4.02) | 3758.63 (35.63) | 29860.5 (39.84) | NR |
| 1 | 15 | 10 mg BID (N= 1) | 1 | 2.17 | 211.00 | 1010.26 | 53.10 |
|  |  | 20 mg BID (N= 14) | 5 | 2.98 (1.97-4.07) | 325.39 (33.72) | 1792.31 (34.99) | 101.84 (51.75) n= 13 |
|  |  | 40 mg BID (N= 32) | 12 | 2.11 (1.97-4.03) | 717.62 (56.95) | 3785.92 (53.01) | 244.13 (82.51) n= 29 |
|  |  | 80 mg BID (N= 12) | 11 | 2.13 (2.00-3.00) | 2046.19 (28.98) | 10908.1 (41.04) | 911.65 (67.82) |
|  |  | 150 mg BID (N= 13) | 12 | 2.00 (1.92-4.07) | 3573.10 (42.59) | 19872.5 (55.49) | 1767.52 (65.08) n= 11 |
|  |  | 160 mg BID (N= 11) | 11 | 2.17 (1.00-3.92) | 4092.58 (38.85) | 21912.0 (40.85) | 1979.34 (51.67) |
|  |  | 200 mg BID (N= 62) | 23 | 2.10 (0.50-4.00) | 5433.76 (32.87) | 30273.7 (30.97) | 2944.69 (43.95) n= 53 |
|  |  | 80 mg QD (N= 18) | 16 | 2.15 (1.02-4.37) | 1510.53 (35.78) | 13702.6 (33.74) | 208.23 (44.93) n= 17 |
|  |  | 120 mg QD (N= 22) | 20 | 2.03 (0.98-4.00) | 2299.93 (31.41) | 19721.2 (32.90) | 330.79 (43.54) n= 20 |
|  |  | 200 mg QD N= 12) | 8 | 2.05 (1.00-4.00) | 4005.44 (35.97) | 38154.5 (36.72) | 614.83 (103.29) n= 9 |
| 2 | 1 | 10 mg BID (N= 1) | 1 | 2.00 | 169.00 | 921.78 | 45.50 |
|  |  | 20 mg BID (N= 14) | 12 | 2.03 (1.25-6.00) n= 13 | 394.00 (89.18) n= 13 | 1935.53 (77.27) | 98.59 (91.56) n= 13 |
|  |  | 40 mg BID (N= 32) | 30 | 2.01 (1.00-6.00) | 793.26 (48.92) | 3967.03 (49.64) | 262.50 (67.53) |
|  |  | 80 mg BID (N= 12) | 11 | 2.02 (1.50-3.97) | 2030.35 (39.60) | 10982.0 (45.13) | 851.64 (75.49) |
|  |  | 150 mg BID (N= 13) | 10 | 2.00 (1.97-3.00) | 3298.19 (48.53) | 17711.4 (59.59) | 1609.16 (41.92) |
|  |  | 160 mg BID (N= 11) | 10 | 2.02 (1.87-3.03) | 4571.36 (35.26) | 24221.3 (40.82) | 2382.67 (45.15) |
|  |  | 200 mg BID (N= 62) | 54 | 2.00 (0.90-7.03) | 5641.84 (39.86) | 29924.6 (41.27) | 2715.38 (57.65) n= 55 |
|  |  | 80 mg QD (N= 18) | 17 | 2.00 (0.95-4.10) | 1780.98 (23.34) | 15001.3 (28.27) | 193.26 (39.58) n= 16 |
|  |  | 120 mg QD (N= 22) | 20 | 2.00 (1.00-3.17) | 2453.12 (28.12) | 20728.8 (32.00) | 303.31 (47.02) |
|  |  | 200 mg QD (N= 12) | 10 | 2.02 (2.00-3.00) | 4161.10 (46.09) | 36196.4 (41.76) | 444.32 (67.73) |

AUC_last_, area under the curve up to the last measurable concentration; BID, twice daily; C_max_, maximum concentration; CV, coefficient of variation; QD, once daily; T_last_, time of last measurable concentration; T_max,_ time to maximum concentration.

^a^ Data are based on the analysis of all patients with chronic myeloid leukemia in chronic phase or accelerated phase with or without *BCR::ABL1*^T315I^ who received asciminib monotherapy. CV% =  sqrt (exp (variance for log transformed data) −1)*100.

^b^ T_last_ for BID was 8 or 12 hours depending on the time when the last pharmacokinetics sample was collected; for QD, T_last_ was 24 hours. 

Figure S1. Study design

ALL, acute lymphoblastic lymphoma; BP, blast phase; CML, chronic myeloid leukemia; DAS, dasatinib; ECOG, Eastern Cooperative Oncology Group; IMA, imatinib; NIL, nilotinib; Ph+, Philadelphia chromosome positive.

^a^ Patients with CML-CP with *BCR::ABL1*^T315I^ and relapsed disease were eligible after ≥ 1 prior TKI if no other effective therapy was available. Patients with CML-BP or Ph+ ALL who had a cytopathologically confirmed diagnosis and were relapsed or refractory to at least one prior TKI or intolerant of TKIs were eligible.

^b^ Recommended dose for expansion has not been determined and no dose-expansion cohort was opened, but 5 additional patients with CML-BP/Ph+ ALL were enrolled in an enrichment cohort at 200 mg BID. 

^c^ Dose expansion of asciminib + imatinib is being assessed in a separate phase 2 study (NCT03578367).

Figure S2. Duration of study treatment vs most recent prior TKI by number of previous TKIs in evaluable^a^ patients

 

TKI, tyrosine kinase inhibitor.	

^a^ Three patients with 1 prior TKI of 115 total patients were omitted. Of the 112 remaining patients, 80 had available information regarding the duration of treatment of their last prior TKI. 

Figure S3. Cumulative rate of *BCR::ABL1*^IS^ ≤ 1% in evaluable^a^ patients by starting dose 

BID, twice daily; IS, International Scale; QD, once daily.

^**a**^ Included all patients with evaluable transcripts not expressing an atypical, p190, or unknown transcripts and not having a missing evaluation at screening.

**References**

1.	Mauro MJ, Hughes TP, Kim DW, Rea D, Cortes JE, Hochhaus A, et al. Asciminib monotherapy in patients with CML-CP without BCR::ABL1 T315I mutations treated with at least two prior TKIs: 4-year phase 1 safety and efficacy results. Leukemia. 2023.
